# Soluble Epoxide Hydrolase Inhibition Regulates Septoclast Activity to Promote Long Bone Growth and Fracture Healing by Enhancing Endothelial‐to‐Mesenchymal Notch Signalling

**DOI:** 10.1111/cpr.70249

**Published:** 2026-06-15

**Authors:** Weixian Chen, Liangliang Liu, Xumeng Wang, Juanjuan Li, Jingyi Zhang, Xiaoli Shi, Qing Sun, Xu Chang, Jing Zhang, Jie Zhao, Fu Wang

**Affiliations:** ^1^ School of Stomatology Dalian Medical University Dalian China; ^2^ Yunnan Key Laboratory of Stomatology and Department of Dental Research The Affiliated Stomatology Hospital of Kunming Medical University Kunming China; ^3^ Academician Laboratory of Immune and Oral Development & Regeneration Dalian Medical University Dalian China; ^4^ National‐Local Joint Engineering Research Center for Drug‐Research and Development (R&D) of Neurodegenerative Diseases, Dalian Medical University Dalian China; ^5^ Stomatological Hospital, Dalian Medical University Dalian China

**Keywords:** endochondral ossification, fatty acid metabolism, fracture healing, septoclasts, soluble epoxide hydrolase, TPPU

## Abstract

Endochondral ossification is essential for the development of appendicular bones, physiological bone remodelling and fracture healing. Recent studies have identified mesenchymal stromal cell‐derived FABP5^+^ septoclasts (SCs) as key mediators for the growth and repair of long bones, particularly in cartilage matrix degradation and growth plate remodelling via the secretion of matrix metalloproteinases. Our previous study has shown that soluble epoxide hydrolase (sEH) inhibitor, 1‐trifluoromethoxyphenyl‐3‐(1‐propionylpiperidin‐4‐yl) urea (TPPU), promotes long bone growth and bone repair by enhancing H‐type vessel‐coupled osteogenesis. However, whether TPPU treatment regulates SC activity, thereby promoting long‐bone growth and fracture healing, remains unclear. Here, our in vitro and in vivo results showed that TPPU treatment promoted long‐bone growth in newborn mice and regulated the hypertrophic layer in the growth plate, with a reduced ratio of hypertrophic cartilage (HC) to proliferative cartilage (PC) width. Furthermore, TPPU treatment enhanced SC activity, as evidenced by elevated expression of MMP9 and FABP5 in the metaphysis near the growth plate. Simultaneously, TPPU induced FABP5^+^ SC‐like cells to degrade chondrocytes in co‐cultured human umbilical vein endothelial cells (HUVECs) and human dental pulp stem cells (hDPSCs). Mechanistically, TPPU enhanced the crosstalk of co‐cultured HUVECs and hDPSCs to activate the NOTCH signalling pathway in hDPSCs by upregulating HIF‐1α expression in HUVECs. Furthermore, TPPU enhanced fracture healing by inducing more FABP5^+^ SCs and MMP9 secretion at the fracture site. Collectively, these findings highlight sEH as a promising therapeutic target that regulates endochondral ossification through inducing SC activity, offering new opportunities for bone development and repair.

## Graphical abstract

The sEH inhibitor TPPU upregulates endothelial HIF‐1α, activating the NOTCH signalling pathway between endothelial cells and mesenchymal stem cells. This promotes the differentiation and formation of FABP5^+^ septoclasts, enhances their participation in endochondral ossification, and ultimately improves longitudinal bone growth and fracture healing.

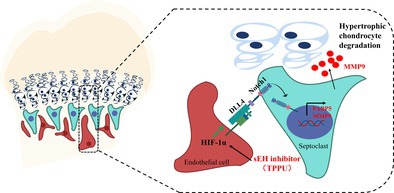

## 1 Introduction

Bones provide structural support and protection, playing critical roles in growth, movement and blood cell production. Skeletal formation is principally governed by endochondral and intramembranous ossification. Endochondral ossification predominantly facilitates long bone formation and supports injury repair, whereas intramembranous ossification primarily forms maxillofacial bones [1]. During endochondral ossification, a cartilaginous framework initially forms and is subsequently and progressively substituted by bone tissue for bone development and regeneration [2]. This process primarily occurs within the growth plate, where hypertrophic chondrocytes (HC) resorption is essential for recruiting cells, facilitating blood vessel invasion and initiating ossification [3, 4]. Disruption of this process can lead to developmental disorders, such as bone dysplasia [5]. During puberty, the growth plate gradually thins and eventually closes, forming a fixed epiphyseal line and halting further longitudinal bone growth [6]. Cartilage resorption involves angiogenic endothelial cells (ECs), osteoclasts and perivascular cells [7]. Previous research has primarily attributed cartilage resorption to osteoclasts—multinucleated cells derived from bone marrow mononuclear cells that degrade mineralised bone [8]. A recent study has identified another special type of ECs at the bone/cartilage interface, termed vessel‐associated osteoclast (VAO) for cartilage degradation [9].

Recently, septoclasts (SCs) have been identified as chondroclasts at the cartilage‐bone junction beneath the growth plate of long bones. These cells originate from mesenchymal stromal lineages and are distinguished by the expression of epidermal fatty acid–binding protein (E‐FABP/FABP5) [10]. According to multiple previous studies, SCs originate from a lineage distinct from monocytes/macrophages, osteoclasts, osteoblasts and ECs, characterised by extensions directed toward the transverse septum [10, 11, 12]. These cells actively facilitate blood vessel invasion into the cartilage, aiding in the degradation and resorption of cartilage septum [13]. SCs specifically secrete matrix metalloproteinases (MMPs), including MMP9, MMP11, MMP13 and MMP14, which synergistically degrade the cartilage matrix and support vascular invasion [10]. MMP9, a key regulator of angiogenesis and osteogenesis, is highly expressed around SCs at the cartilage‐bone boundary. It serves as the most abundant and functionally central MMP in SCs, initiating cartilage matrix degradation, promoting vascular sprouting and being directly regulated by endothelial DLL4‐Notch signalling; therefore, it represents the most sensitive and reliable marker for SC presence, activity and functional status [10, 14]. During ossification, increased numbers of FABP5^+^ and MMP9^+^ SCs correlate with dynamic remodelling of the growth plate [15]. In contrast, MMP9 expression in SCs declines with age, suggesting its critical role in cartilage degradation during bone growth [13]. MMP13 plays a complementary role in cartilage degradation by cleaving type II collagen and aggrecan [16]. The coordinated action of MMP9 and MMP13 ensures efficient cartilage resorption during bone formation. Notch signalling is recognised as a key pathway governing the crosstalk between ECs and SCs. Genes associated with the Notch pathway, such as *Hey1* and *Heyl*, are highly expressed in SCs, while *Notch1* and *Notch4* are abundantly expressed in ECs. Direct interactions occur between DLL4^+^ ECs and HEY1^+^ SCs [10].

Soluble epoxide hydrolase (sEH) mediates the metabolic conversion of short‐chain fatty acid epoxyeicosatrienoic acids (EETs) into their inactive dihydroxyeicosatrienoic acids (DHETs) forms [17, 18]. 1‐trifluoromethoxyphenyl‐3‐(1‐propionylpiperidin‐4‐yl) urea (TPPU), a specific sEH inhibitor, prevents EETs inactivation, thereby increasing EETs levels, which exert anti‐inflammatory, pro‐angiogenic and immune regulation in various tissues [19, 20, 21]. In the context of bone, elevated EETs have been shown to promote osteoblast differentiation and angiogenesis [22]. Our previous study confirmed that TPPU promotes long bone formation by coupling type H angiogenesis and osteogenesis through activation of the SLIT3/HIF‐1α pathway [23]. Therefore, we hypothesise that the regulatory effects of EET elevation induced by TPPU on SCs are mediated indirectly through endothelial‐mesenchymal signalling, rather than through direct regulation of SCs themselves. In promoting osteogenic‐angiogenic coupling, the PI3K/Akt and MAPK pathways play core roles in angiogenesis, osteogenic differentiation and cell–cell crosstalk [24]. HIF‐1α couples angiogenesis and osteogenesis via the VEGF/AKT/mTOR signalling pathway, significantly enhancing osseointegration and new bone formation [25]. Notch signalling serves as a key molecular bridge between ECs and mesenchymal stem cells (MSCs) for osteogenic‐angiogenic coupling. ECs regulate MSCs fate through Notch‐dependent signalling (e.g., Jagged‐1 ligand), promoting their simultaneous differentiation toward osteogenic and angiogenic lineages [26, 27]. However, the mechanism by which EETs levels affect SCs differentiation and activity through interactions with MSCs remains unclear.

Here, we found that TPPU, an sEH inhibitor, enhances the formation of FABP5^+^ SCs through the Notch signalling pathway, thereby regulating endochondral ossification in long bones. Additionally, TPPU significantly improves fracture healing, a process closely linked to increased SC activity at the fracture site. These findings reveal a novel mechanism underlying bone development and repair via endochondral ossification and suggest potential therapeutic targets for clinical fracture treatment.

## 2 Materials and Methods

### 2.1 Animals

Eight‐week‐old C57BL/6J mice were obtained from the Experimental Animal Center of Dalian Medical University. All animal studies complied with the ARRIVE 2.0 guidelines and were approved by the Animal Care and Use Committee of Dalian Medical University (No. AEE23141). A total of 18 eight‐week‐old mice were used in this study, including pregnant female mice (*n* = 3 per group, 6 in total) for the maternal treatment experiment and adult male mice (*n* = 6 per group, 12 in total) for the fracture model experiment. The mice were randomly assigned to either the TPPU‐treated group or the vehicle control group. The dosage of TPPU (3 mg/kg, dissolved in PEG400) was determined based on previous studies from other research groups as well as our own [23, 28, 29, 30]. Briefly, mice in the TPPU+ group received daily gavages of TPPU at 3 mg/kg, dissolved in PEG400, while the vehicle control group was administered the same volume of PEG400.

For the maternal experiment, pregnant mice received daily oral gavage of TPPU or vehicle control from day 14 of pregnancy until 1 week postpartum. Offspring were used for subsequent analyses, with approximately 6 neonatal mice per group.

### 2.2 Cell Culture

Human umbilical vein endothelial cells (HUVECs) (ATCC, Virginia, USA) were maintained in endothelial cell medium (ECM, ScienCell, California, USA) supplemented with 1% endothelial cell growth supplement (ECGS, ScienCell), 10% fetal bovine serum (FBS, Gibco, New York, USA), 100 U/mL penicillin (Solarbio, Beijing, China) and 100 μg/mL streptomycin (Solarbio).

The isolation and cultivation of human dental pulp stem cells (hDPSCs) followed an established protocol [31]. Briefly, dental pulp tissues were obtained from teeth extracted for orthodontic purposes at the Stomatological Hospital of Dalian Medical University, with informed consent and ethical approval (No. 2022001). The tissues were minced and digested to generate a single‐cell suspension. The isolated cells were then cultured in DMEM/F12 medium (Gibco) containing 10% FBS, 1% penicillin/streptomycin, maintained at 37°C in a humidified atmosphere with 5% CO_2_ for 3–5 days until reaching 80% confluence.

hDPSCs were seeded at a density of 8000 cells/cm^2^. The stem cell phenotype of hDPSCs was confirmed based on previously established characterisation, including multipotent differentiation potential and flow cytometric analysis reported in our previous studies [32]. For co‐culture experiments, HUVECs and hDPSCs were cultured under direct contact conditions at a ratio of 1:1, with a total seeding density of 10,000 cells/cm^2^.

Primary chondrocytes were harvested from the femoral head of 6‐day‐old C57BL/6J mice via trypsin digestion, as described [33]. After filtration and centrifugation, cells were seeded at a density of 8000 cells/cm^2^ in DMEM/F12 supplemented with 10% FBS.

### 2.3 RT–qPCR

Following the extraction of total RNA with RNAkey Reagent (SM139‐02, SEVEN, Beijing, China), cDNA was synthesised and quantitative PCR was carried out using the SevenFast Two Step RT‐qPCR Kit (SRQ‐01, SEVEN). All primers were designed using Primer Premier software to ensure specificity for target genes and synthesised by Sangon Biotech (Shanghai, China). Analysis of relative gene expression was conducted through normalising to *GAPDH* expression with the 2^−ΔΔ*Ct*^ method. Corresponding primer sequences can be found in Table S1.

### 2.4 Cell Transfection

HUVECs were transfected with HIF‐1α knockdown virus (GiKai gene, Shanghai, China) according to the instructions provided by Genechem. After transfection, cells were selected using medium containing 1 μg/mL puromycin (Coolaber, Shanghai, China) to eliminate non‐transfected cells. The sequences for targeting HIF‐1α were provided in Table S2. The transfected HUVECs were subsequently used in co‐culture experiments with hDPSCs.

### 2.5 Western Blotting

Proteins were extracted, quantified and analysed by Western blot. Briefly, lysates (RIPA buffer with inhibitors) were prepared, and 15 μg of protein from each sample was resolved by SDS‐PAGE and transferred onto PVDF membranes. Following a blocking step, the membranes were incubated sequentially with primary antibodies and HRP‐conjugated secondary antibodies (1:8000, ZF‐0317, ZSGB‐BIO, Beijing, China). Signals were detected with an ECL kit (Tanon, Shanghai, China) and imaged on a Bio‐Rad VersaDoc system. The primary antibodies used included anti‐HIF‐1α (1:1000, ab2185, Abcam, Cambridge, UK), anti‐FABP5 (1:1000, 12348‐1‐AP, Proteintech, Wuhan, China), anti‐DLL4 (1:1000, ER1706‐29, HuaBio, Zhejiang, China), anti‐NOTCH1 (1:1000, ET1606‐55, HuaBio), anti‐GAPDH (1:8000, ET1601‐4, HuaBio).

### 2.6 Histology, Immunohistochemistry and Immunofluorescence

Tissue specimens were fixed in 4% paraformaldehyde, paraffin‐embedded and sectioned at a thickness of 5 μm for subsequent analyses. Histochemical evaluation was conducted with H&E staining (G1120, Solarbio), TRAP staining (G1050, ServiceBio, Wuhan, China), Safranin O‐Fast Green (G1371, Solarbio) or Alcian Blue (G1560, Solarbio) staining as required. Growth plate analysis was performed on standardised longitudinal sections. The hypertrophic (HC) and proliferative (PC) zones were defined based on established morphological criteria, and their relative lengths were measured to calculate the HC/PC ratio. In addition, the hypertrophic cartilage region was quantified. All measurements were performed in a blinded manner on multiple sections per sample using ImageJ software.

For immunohistochemistry (IHC), sections were dewaxed and rehydrated. After blocking, they were incubated with primary antibodies targeting FABP5 (1:200) or MMP9 (1:200, ab38898, Abcam) overnight at 4°C. Following washes, the signals were detected using horseradish peroxidase (HRP)‐conjugated secondary antibodies (PV9000, ZSGB‐BIO) for 1 h at room temperature, visualised with diaminobenzidine (DAB) chromogen (DA1010, Solarbio) and haematoxylin counterstain (Solarbio).

For immunofluorescence (IF), sections were incubated with primary antibodies targeting EMCN (1:100, ab106100, Abcam), FABP5 (1:200), MMP9 (1:200), MMP13 (1:200, YP‐rAb‐18052, UpingBio), DLL4 (1:200) or F‐actin (1:500, ab205, Abcam) overnight at 4°C. After washing, sections were probed with appropriate fluorescent dye‐conjugated secondary antibodies (ab150077, ab150079, ab6785, ab6786, Abcam) for 1 h at room temperature, counterstained with DAPI, and imaged using an inverted fluorescence microscope. Apoptosis was assessed using TUNEL assay (BrightGreen Apoptosis Detection Kit, A112, Vazyme, Nanjing, China).

### 2.7 Mouse Tibia Fracture

A standardised tibial closed transverse fracture model was created in 8‐week‐old C57BL/6J mice (6 female and 6 male). Anaesthesia was induced via intraperitoneal injection of ketamine hydrochloride (60 mg/kg) and xylazine hydrochloride (10 mg/kg), after which the right hind limb's knee joint was surgically exposed. A standardised closed transverse fracture was created at the mid‐diaphysis of the tibia using a three‐point bending device, followed by intramedullary fixation with a stainless‐steel needle. All fractures were generated at a consistent anatomical location using the same device, and all surgical procedures were performed by the same investigator to ensure reproducibility. Finally, the surgical incision was sutured. Mice in the TPPU‐treated group received TPPU (3 mg/kg) by gavage every other day for 2 weeks, while control mice received an equivalent volume of vehicle. Mice were euthanised and sampled at the third week post‐surgery. The fractured tibiae were then harvested for radiographic assessment and subsequently decalcified in 10% EDTA solution for subsequent IHC analysis.

### 2.8 Micro‐CT

Micro‐CT imaging was conducted on a Bruker Skyscan 1276 system under standardised settings (voxel size: 6.534165 μm; medium resolution; 70 kV, 200 μA; 1 mm Al filter; integration time: 350 ms), with density calibration against a CaHA phantom. Subsequent processing and analysis involved image reconstruction (NRecon v1.7.4.2), 3D model generation (CTvox v3.3.0 via grayscale distance transformation), and detailed 2D/3D morphometric evaluation of bone microstructure within a defined ROI using CT Analyser software (v1.20.3.0).

### 2.9 Statistical Analysis

The value of n represents the number of independent biological replicates unless otherwise stated. All statistical analyses were conducted using SPSS version 26.0. Data, derived from at least three independent experiments, are presented as means ± SEM. After confirming normal distribution via normality testing, group means were compared using *t*‐tests or one‐way ANOVA, as appropriate. Differences were considered statistically significant at *p* < 0.05. All analyses were performed in a blinded manner where applicable.

## 3 Results

### 3.1 TPPU Promotes Endochondral Ossification and Increases SCs Number in Newborn Mice

**FIGURE 1:**
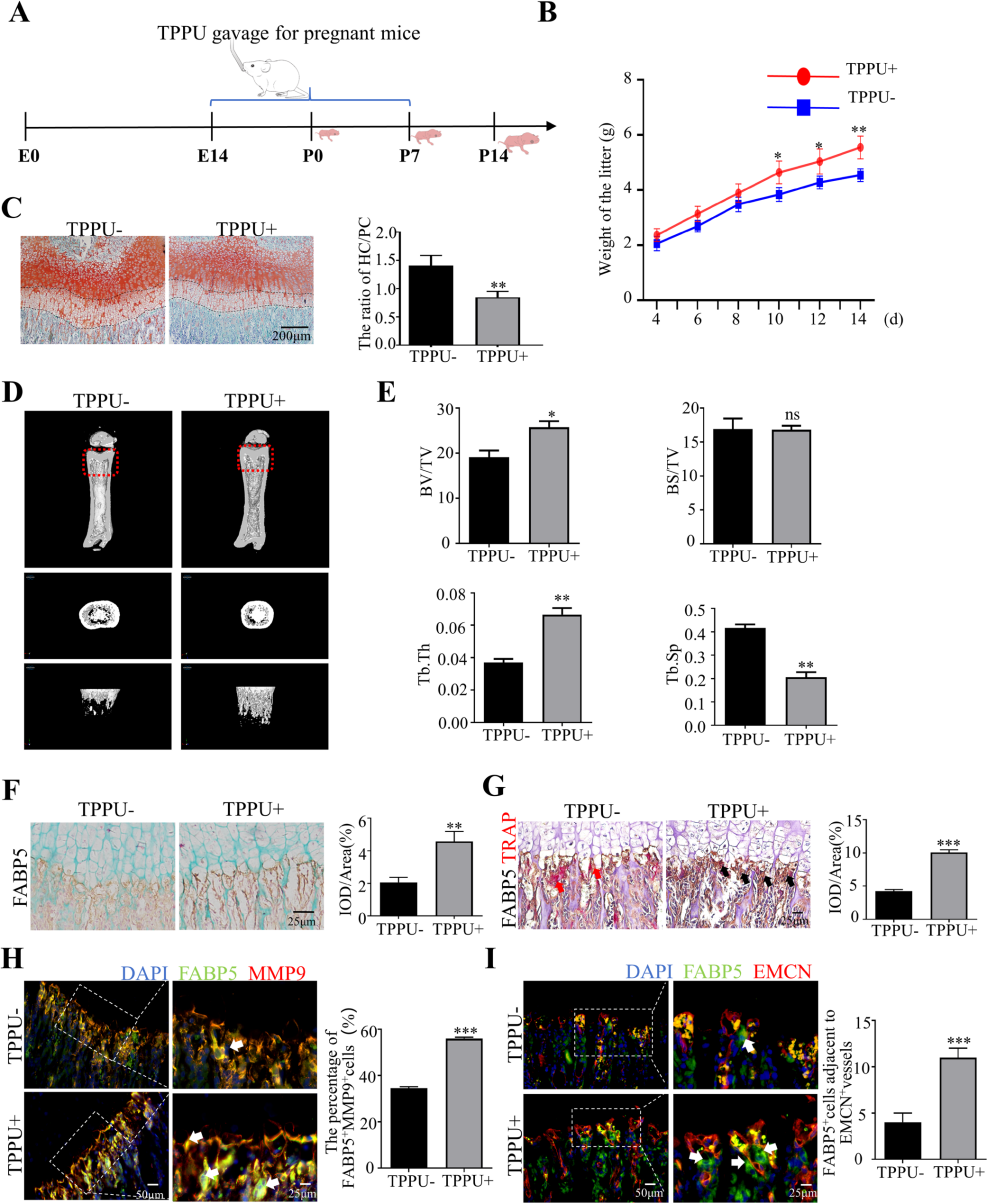
TPPU promotes long bone growth, enhances endochondral ossification, and increases the population of SCs at the COJ in newborn mice. (A) Schematic representation of TPPU gavage to pregnant mice for analysis of offspring long bone growth. (B) Body weight of offspring from pregnant mice treated with or without TPPU. (C) Safranin O‐Fast Green staining of femurs from 2‐week‐old pups. Quantitative analysis of the ratio of HC/PC. The HC and PC regions were measured from longitudinal sections based on morphological criteria. (D, E) Structural and quantitative assessment of femurs from 2‐week‐old pups via micro‐CT. (F, G) Representative IHC images showing FABP5 expression in the growth plate–adjacent metaphysis of 2‐week‐old neonatal mice, following (F) Alcian Blue and (G) TRAP staining. Scale bar = 25 μm. (H) FABP5^+^ MMP9^+^ SCs (white arrows) were observed near the growth plate. The left scale bar = 50 μm, the right scale bar = 25 μm. (I) FABP5^+^(green) SCs (white arrows) located near EMCN^+^ vessel buds (red). The left scale bar = 50 μm, the right scale bar = 25 μm. *p < 0.05, **p < 0.01. *n* = 6.

We previously established that TPPU acts via expanding the type H vessels to drive the critical coupling between angiogenesis and osteogenesis, accelerating long bone growth and defect repair. However, its impact on endochondral ossification remains unclear. To explore this, we orally administered TPPU to female mice from Day 14 of pregnancy through 1 week post‐delivery to assess its effects on endochondral ossification in the offspring's long bones (Figure 1A). Pups born to TPPU‐treated mothers were heavier than control pups (Figure 1B). Representative images of femurs from pups in the TPPU− and TPPU+ groups are shown in Figure S1A. We evaluated HC resorption by calculating the ratio of hypertrophic chondrocytes (HC) to proliferative chondrocytes (PC) length. Growth plate analysis showed that pups born to TPPU‐treated females had a reduced HC/PC ratio with reduced hypertrophic cartilage region compared to controls. The TPPU‐ group displayed significantly more cartilage remnants in the metaphysis compared to the TPPU+ group (Figures 1C and S1B,C). Micro‐CT analysis further showed that bone mass in metaphysis of femurs was increased in pups born to TPPU‐treated females (Figure 1D). Specifically, Micro‐CT analysis revealed significantly increased bone volume fraction (BV/TV) and trabecular thickness (Tb.Th) but decreased trabecular separation (Tb.Sp). In contrast, the bone surface density (BS/TV) did not differ significantly between the TPPU+ and TPPU− groups (Figure 1E). These results suggest that TPPU may facilitate HC resorption and promote endochondral ossification of long bones.

SCs exclusively express FABP5 and secrete MMP9. We found that TPPU treatment markedly enhanced FABP5 levels in the metaphysis of neonatal mice, with signal especially localised to the chondro‐osseous junction (COJ) (Figure 1F,G). However, FABP5 expression did not differ significantly between the two groups at postnatal Day 0 or Day 7 (Figure S2A). SCs contribute to HC degradation by secreting MMP9, a key regulator of angiogenesis and osteogenesis, which aids in the phagocytosis of HC debris. TPPU treatment increased both the number of SCs and their expression of MMP9 and FABP5 near the growth plate. Co‐immunofluorescence staining revealed FABP5^+^ MMP9^+^ SCs at the chondro‐osseous junction (Figure 1H). These FABP5^+^ SCs were also located in proximity to EMCN^+^ distal vascular buds (Figures 1I and S2B). To further evaluate matrix‐degrading activity associated with SCs, we examined the expression of MMP13. Immunofluorescence analysis revealed increased MMP13 expression in TPPU‐treated groups both in vivo and in vitro. In vivo, co‐staining of FABP5 and MMP13 in the metaphyseal region of 2‐week‐old neonatal mice demonstrated a higher proportion of MMP13^+^ cells within the FABP5^+^ population in TPPU‐treated samples (Figure S2C). Similarly, in the co‐culture system, TPPU treatment increased the proportion of MMP13^+^ cells (Figure S2D). These findings further support enhanced matrix remodelling activity associated with SCs.

### 3.2 TPPU Induces FABP5 ^+^ Cells in Co‐Cultured HUVECs and hDPSCs to Degrade Chondrocytes

**FIGURE 2:**
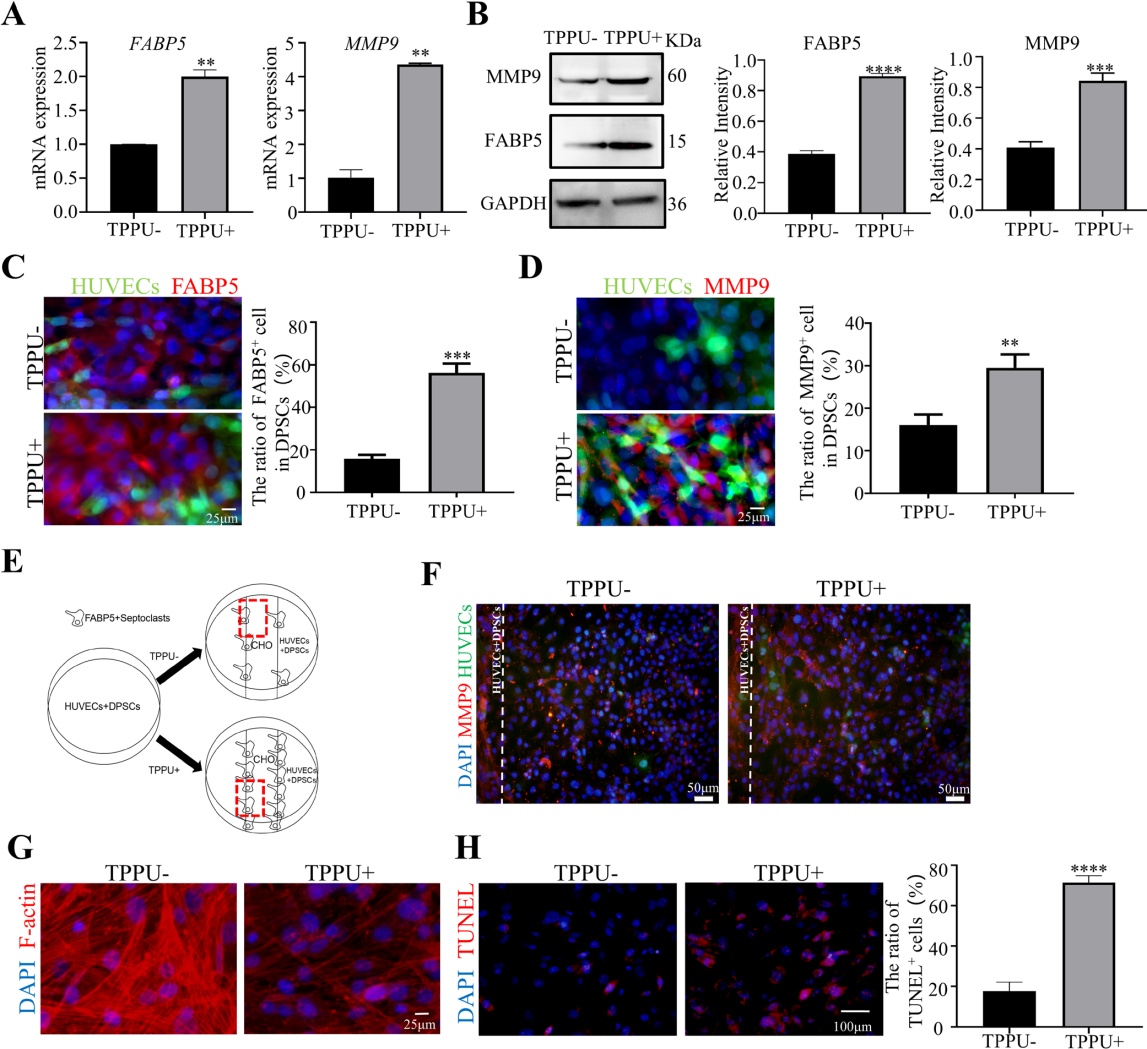
TPPU induces FABP5^+^ cells in co‐cultured cells to degrade chondrocytes. (A, B) Analysis showing FABP5 and MMP9 expression by RT‐qPCR and Western Blot in co‐cultured HUVECs and hDPSCs (*n* = 3 samples from three independent experiments). Figure S5 for full‐length blots/gels. (C) Representative image showing FABP5^+^ cells (red) in co‐cultured HUVECs (labelled with GFP, green) and hDPSCs (*n* = 3 samples of three independent experiments). (D) Representative image showing MMP9 expression (red) in co‐cultured HUVECs (green) and hDPSCs. Scale bar = 25 μm. (E) Schematic representation of chondrocytes cultured with mixed HUVECs (green) and hDPSCs. (F) Immunofluorescence assays for MMP9 in dashed boxes. Scale bar = 50 μm. (G) F‐actin showing the cytoskeleton of chondrocytes treated with co‐cultured medium. Scale bar = 25 μm. (H) TUNEL assay of chondrocytes cultured in conditional osteogenic medium derived from co‐cultured cells. Scale bar = 100 μm. **p < 0.01, ***p < 0.001, ****p < 0.0001. *n* = 3.

Our earlier research confirmed that TPPU promotes the formation of type H ECs in co‐cultured HUVECs and hDPSCs (co‐cultured cells) through the coupling of osteogenesis‐angiogenesis in vitro. We further investigated whether TPPU also induced SC‐like cells in addition to promoting osteogenesis‐angiogenesis coupling in these co‐cultured cells. Consistent with our in vivo results, TPPU treatment under osteogenic differentiation conditions resulted in a marked upregulation of FABP5 and MMP9 expression at the transcriptional and translational levels in co‐cultured cells (Figure 2A,B; Uncropped blot of Figure 2B for FABP5 and MMP9 see Figure S5). This induction was further confirmed by immunofluorescence in GFP^+^ HUVEC/hDPSC co‐cultures where TPPU enhanced the expression of FABP5 and MMP9 in hDPSCs (Figure 2C,D). To assess the functional impact on cartilage remodelling, chondrocytes were added to a scratch wound in the same co‐culture system. TPPU enhanced chondrocyte degradation, an effect correlated with elevated MMP9 expression in the chondrocyte regions (Figure 2E,F). TPPU also disrupted the chondrocyte cytoskeleton, reducing F‐actin expression in chondrocytes cultured in conditional osteogenic medium derived from co‐cultured cells (Figure 2G). Moreover, TPPU treatment promoted chondrocyte apoptosis (Figure 2H). However, TPPU had little effect on treating HUVECs (Figure S3A–C; Uncropped blot of Figure S3A for DLL4, NOTCH1 and FABP5 see Figure S11) or hDPSCs alone (Figure S3D–F; Uncropped blot of Figure S3D for NOTCH1, MMP9 and FABP5 see Figure S12). Our data suggest that TPPU promotes chondrocyte degradation by inducing a population of FABP5^+^ mesenchymal cells within the co‐culture system, depending on endothelial‐mesenchymal cells crosstalk.

### 3.3 TPPU Activates NOTCH Signalling to Increase FABP5 ^+^ SCs in Co‐Cultured Cells

**FIGURE 3:**
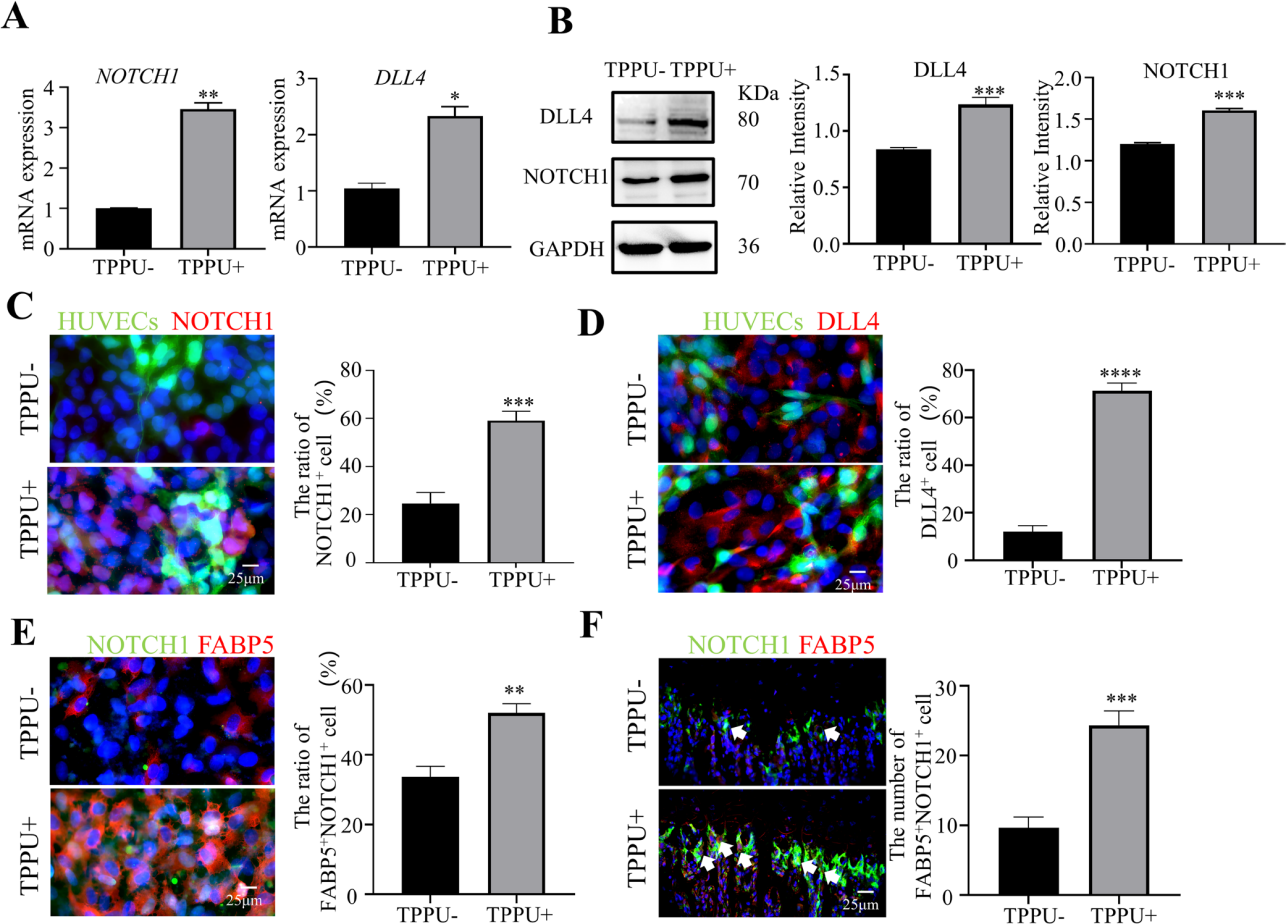
TPPU activates NOTCH signalling to increase FABP5^+^ SCs in co‐cultured cells. (A) mRNA levels of *NOTCH1* and *DLL4* in co‐cultured cells. (B) Expression of NOTCH1 and DLL4 in co‐cultured cells. Figure S6 for Full‐length blots/gels. (C, D) Immunofluorescence assays for NOTCH1 (Red), DLL4 (Red), in co‐cultured HUVECs (green) and hDPSCs. Scale bar = 25 μm. (E) Immunofluorescence assays for NOTCH1 (green) and FABP5 (red) in co‐cultured cells. (F) Expression of NOTCH1 (green) and FABP5 (red) in the metaphysis. *p < 0.05, **p < 0.01, ***p < 0.001, ****p < 0.0001. *n* = 3.

It is well established that ECs drive the differentiation of mesenchymal precursors into SCs via NOTCH pathway activation. Next, we evaluated whether the TPPU activated the NOTCH pathways. Our findings revealed that TPPU administration markedly elevated the levels of NOTCH1 and DLL4, as evidenced by elevated mRNA transcripts and correspondingly higher protein abundance (Figure 3A,B; Uncropped blot of Figure 3B for DLL4 and NOTCH1 see Figure S6). Immunofluorescence staining demonstrated that TPPU upregulated NOTCH1 expression mainly in hDPSCs and upregulated DLL4 expression mainly in HUVECs in co‐cultured cells (Figure 3C,D). Immunofluorescence staining demonstrated that most NOTCH1 expression was co‐localised with FABP5^+^ cells (Figure 3E,F).

**FIGURE 4:**
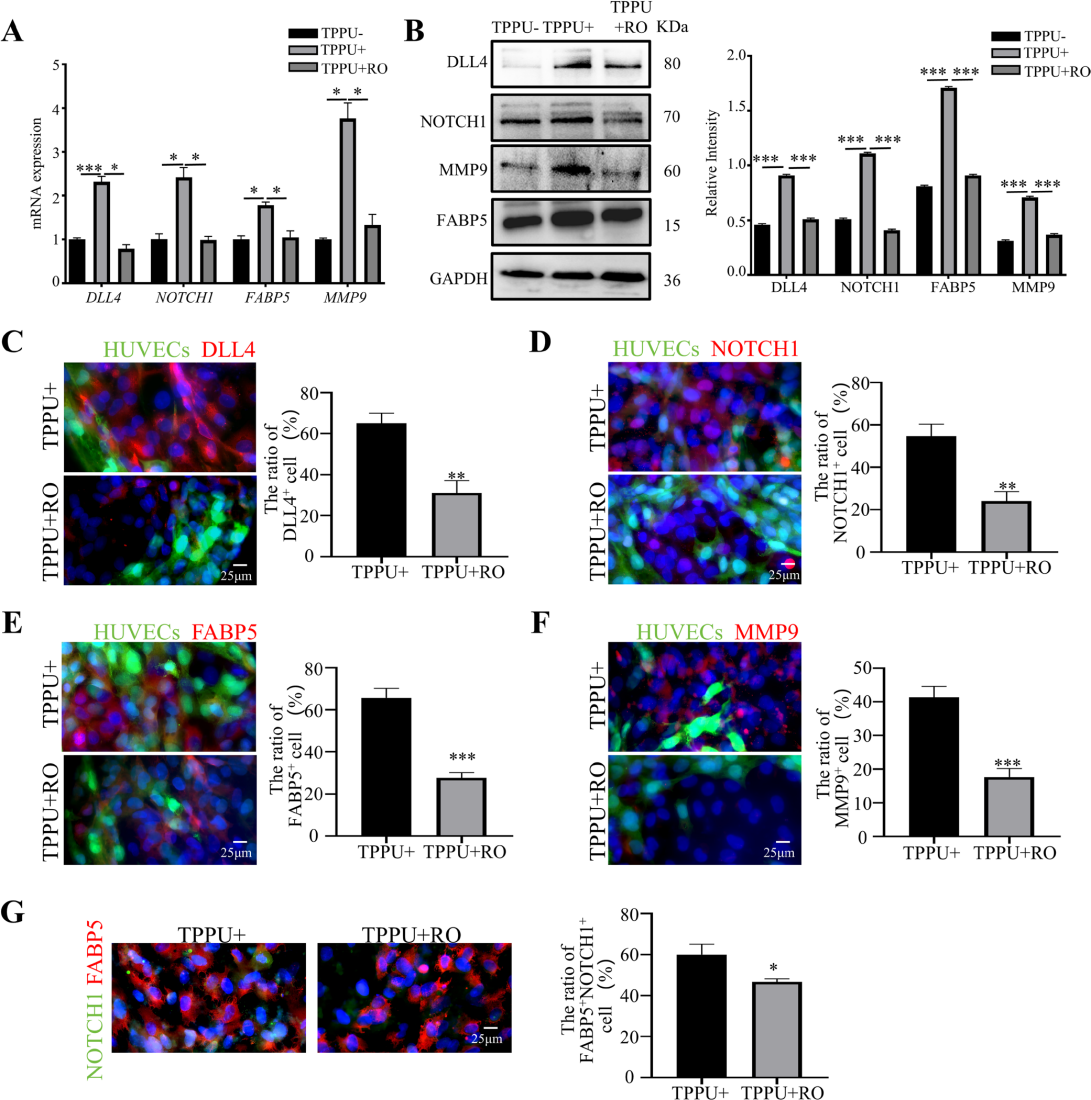
NOTCH1 inhibition blocks the formation of FABP5^+^ SCs in co‐culture cells. (A) mRNA levels of *DLL4, NOTCH1, FABP5 and MMP9* in NOTCH1‐blocked co‐cultured cells. (B) Protein levels of DLL4, NOTCH1, FABP5 and MMP9 in co‐cultured cells. Full‐length blots/gels were provided in Figure S7. (C‐F) Immunofluorescence assays for DLL4, NOTCH1, FABP5 and MMP9 (red) in co‐cultured HUVECs (green) and hDPSCs. (G) Immunofluorescence assays for NOTCH1 (green) and FABP5 (red) in co‐cultured cells. Scale bar = 25 μm. *p < 0.05, **p < 0.01, ***p < 0.001.

When the Notch pathway was blocked using γ‐secretase inhibitor RO4929097 (RO), TPPU treatment downregulated the expression of DLL4, NOTCH1, FABP5 and MMP9 in co‐cultured cells (Figure 4A,B; Uncropped blot of Figure 4B for DLL4, NOTCH1, MMP9 and FABP5 see Figure S7). When HUVECs in the co‐cultured cells were labelled with GFP, immunofluorescence staining confirmed that the Notch pathway blocker RO decreased the levels of NOTCH1, FABP5 and MMP9 in hDPSCs and reduced the expression of DLL4 in HUVECs (Figure 4C–G).

### 3.4 TPPU Activates NOTCH Signalling in Co‐Cultured Cells by Elevating HIF‐1α Expression Within the Endothelial Compartment

**FIGURE 5:**
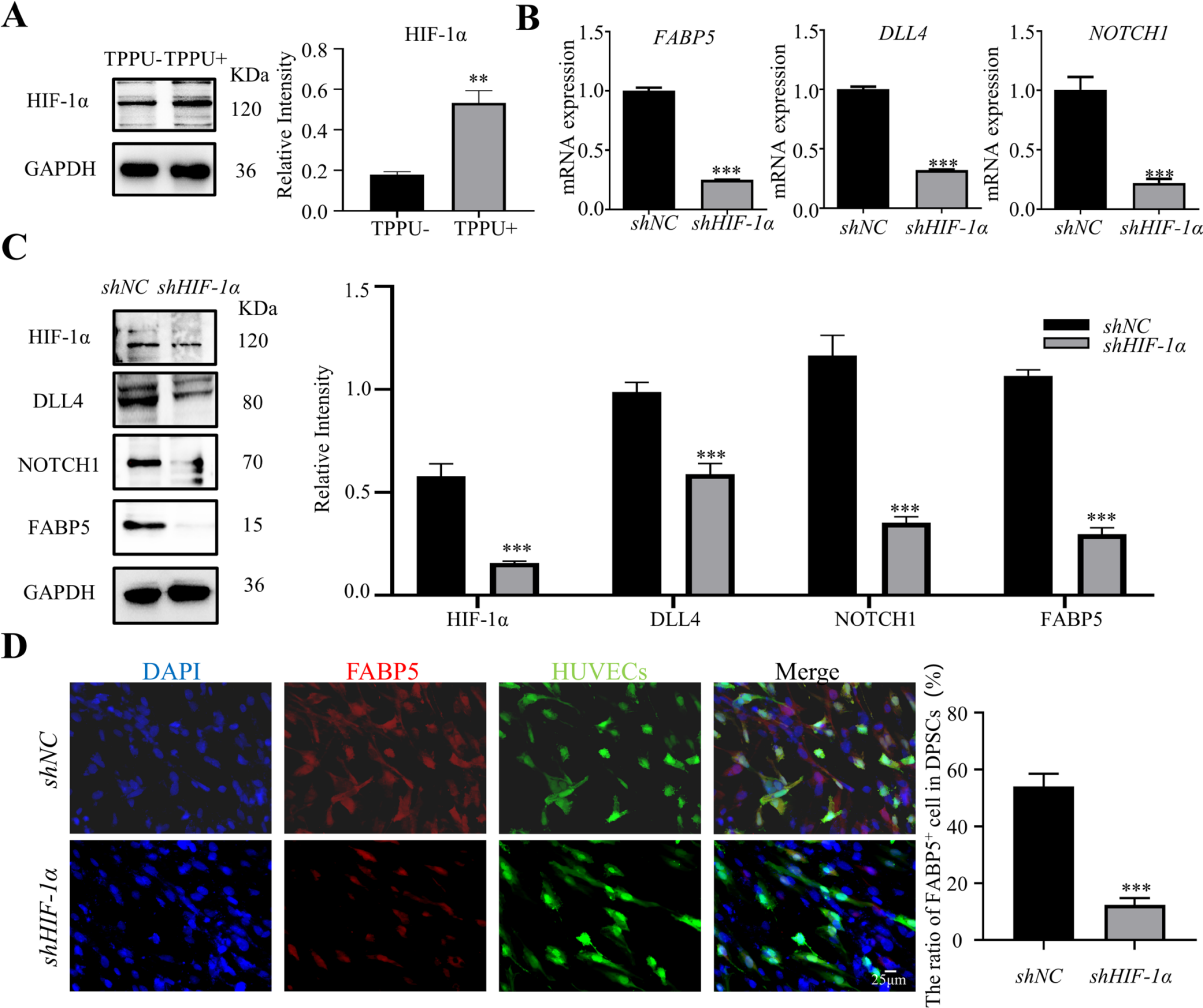
TPPU activates NOTCH signalling by upregulating endothelial HIF‐1α expression in co‐cultured cells. (A) Protein levels of HIF‐1α in co‐cultured cells. Full‐length blots/gels were provided in Figure S8. (B) HIF‐1α knockdown in HUVECs, mRNA expression of *FABP5*, *DLL4* and *NOTCH1* in co‐cultured cells. (C) Protein levels of HIF‐1α, DLL4, NOTCH1 and FABP5 in co‐cultured HUVEC and hDPSCs following HIF‐1α knockdown in HUVECs. Full‐length blots/gels were provided in Figures S9 and S10. (D) Immunofluorescence assays for FABP5^+^ cells (red) in co‐cultured HUVECs (green) and hDPSCs after HIF‐1α knockdown in HUVECs. Scale bar = 25 μm. *p < 0.05, **p < 0.01, ***p < 0.001, ****p < 0.0001. *n* = 3.

HIF‐1α expression in ECs drives type H vessel formation in the metaphyseal region. We previously reported that TPPU induces HIF‐1α expression within the type H endothelial cell population of the murine metaphysis (Figure S4A). Here, we observed a marked upregulation of HIF‐1α protein expression following TPPU treatment in co‐cultured cells (Figure 5A; Uncropped full‐length gels and blots see Figure S8). Upon HIF‐1α knockdown in HUVECs (Figure S4B; Uncropped full‐length gels and blots see Figure S13), a decrease was likewise observed in the mRNA levels of *FABP5*, *DLL4* and *NOTCH1* in co‐cultured cells (Figure 5B). Consistent with this, Western blot analysis further confirmed the suppressive effect of HUVEC‐specific HIF‐1α knockdown on the protein expression of FABP5, DLL4 and NOTCH1 in co‐cultured cells (Figure 5C; Uncropped full‐length gels and blots see Figures S9 and S10). Immunofluorescence staining showed that HUVEC‐specific HIF‐1α knockdown reversed the TPPU‐induced increase in FABP5^+^ cells (Figure 5D). These results suggest that TPPU promotes the cellular interactions between ECs and FABP5^+^ SCs by activating the HIF‐1α/NOTCH signalling axis.

### 3.5 TPPU Promotes Fracture Healing by Increasing FABP5 ^+^ Cells and Enhancing MMP9 Signal in the Fracture Region

**FIGURE 6:**
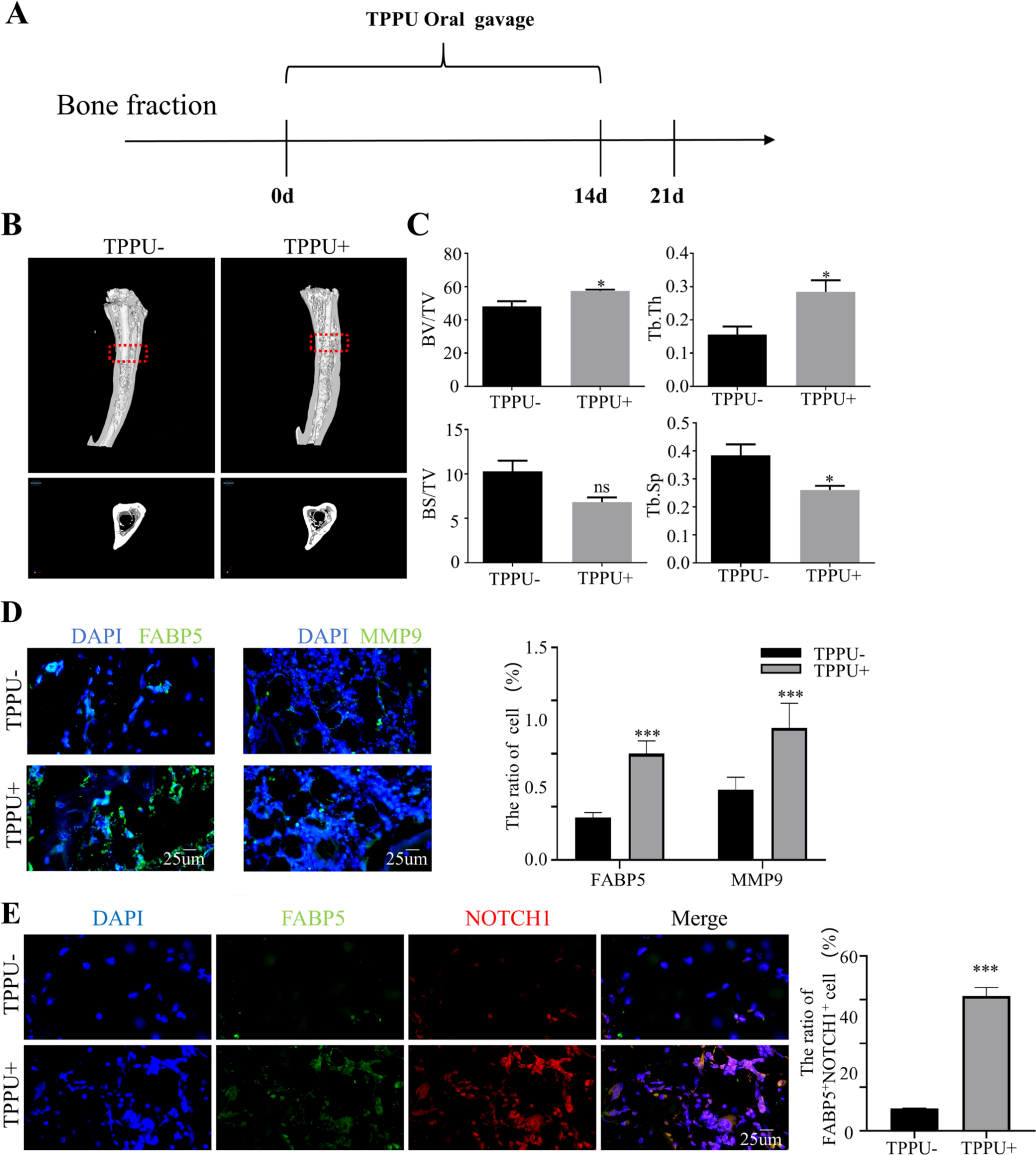
TPPU promotes fracture healing with increased FABP5^+^ cells and high MMP9 signal in the fracture region. (A) Schematic representation of TPPU treatment for bone fracture. (B, C) Representative micro‐CT images and quantitative analysis of tibia fracture at post‐fracture Day 21. *n* = 3. (D) FABP5^+^ cells and MMP9 expression in the fracture region. *n* = 5. The scale bar = 25 μm. (E) Immunofluorescence assays for FABP5 (green) and NOTCH1 (red) in the fracture region. Scale bar = 25 μm. *p < 0.05, **p < 0.01, ***p < 0.001, ****p < 0.0001. *n* = 6.

To further investigate the effect of TPPU on endochondral ossification remodelling, we created a mouse tibial fracture model and administered TPPU by gavage to the experimental group (Figure 6A). TPPU treatment promoted the formation of a more robust trabecular network at the fracture site, as evidenced by significantly greater BV/TV, Tb.N, Tb.Th and reduced Tb.Sp compared to the vehicle‐treated group (Figure 6B,C). Following TPPU treatment, the number of FABP5^+^ SCs was further increased, and MMP9 expression was upregulated at the fracture line (Figure 6D). Additionally, a significant upregulation of NOTCH1 expression was observed in FABP5^+^ SCs localised at the fracture site (Figure 6E).

## 4 Discussion

Endochondral ossification is essential for both embryonic skeletogenesis (including limb, trunk and skull base bones) and fracture repair. Concurrently, longitudinal bone growth is governed by chondrocyte proliferation, with the resultant cartilage matrix providing the template for subsequent ossification [34]. The proper metabolic function of chondrocytes is essential for maintaining optimal bone quality. Successful developmental osteogenesis, bone remodelling and fracture healing all depend on the timely and efficient removal of residual matrix materials and cellular debris. The precise identity of the cells responsible for cartilage resorption remains a contentious issue. Recent studies suggest that FABP5^+^ SCs located at the cartilage–bone boundary in the developing skeleton play a key role in this process [35, 36, 37]. Our results indicate that TPPU‐mediated inhibition of sEH contributes to endochondral ossification by enhancing SC activity through an endothelium‐dependent mechanism, thereby facilitating bone development and fracture healing. This suggests that TPPU regulates SC activity indirectly rather than through direct action on SCs.

In 1995, Lee et al. described the erosion of the septum at the cartilage‐subchondral bone transition in the growth plate, a process that facilitates capillary invasion. Cathepsin B‐high cells are enriched at sites of active capillary growth, where they extend processes rich in cathepsin B‐containing multivesicular dense bodies into the septum, aiding in extracellular matrix breakdown [38]. These cells, which resemble osteoclasts morphologically but lack the osteoclast‐specific marker ED1, are termed septoclasts. Subsequent evidence has shown that *n*‐3 polyunsaturated fatty acids play a key role in regulating long bone growth [39]. E‐FABP‐immunoreactive cells have been identified as SCs under physiological conditions [37].

Recent single‐cell RNA sequencing has revealed that FABP5^+^ cells at the cartilage‐bone boundary originate from MSCs [10]. Notch signalling has been shown to stimulate bone progenitors, enhancing the expression of FABP5 and MMP9. Our results further confirm that TPPU induces FABP5 expression in the mouse metaphysis, an effect mediated by the activation of Notch signalling. These FABP5^+^ cells are clustered near endothelial marker EMCN‐labelled vascular buds, suggesting potential interactions between endothelial and mesenchymal cells. Our findings demonstrate that TPPU activates HIF‐1α signalling in HUVECs, enhancing their interactions with hDPSCs through Notch signalling. HIF‐1α, expressed in ECs, has been identified as a critical activator of H‐type vessel formation in the metaphyseal region. However, the interaction between NOTCH1 and HIF‐1α remains unclear, necessitating further investigation into their relationship.

Fracture healing involves complex biological processes, including subperiosteal osteogenesis and chondrogenesis [40]. Our results demonstrate the potential of TPPU to promote fracture healing by enhancing SC activity, thereby regulating endochondral ossification. In addition to SC‐associated activity, TPPU may also influence other cell types within the fracture microenvironment, including osteoblast‐lineage cells, which could contribute to enhanced matrix remodelling and bone formation. These findings highlight the critical role of SCs in coordinating cartilage remodelling and vascular invasion, and suggest that targeting sEH may represent a promising therapeutic strategy for bone development and repair.

Our previous study demonstrated that TPPU enhances the coupling of angiogenesis and osteogenesis, thereby promoting long bone growth and calvarial bone defect repair in mice. We hypothesise that the regulatory effect of TPPU on SCs may not be direct, but rather indirectly mediated through intercellular signalling between ECs and MSCs. We systematically compared the findings of this study with other recent studies on bone metabolism, including those related to targeting type H vessels to promote bone formation [41, 42, 43, 44]. Although TPPU shows promising efficacy in mouse models, translation to humans requires careful consideration of species differences in metabolism, optimal dosing strategies and potential off‐target effects. The therapeutic effects of TPPU do not arise from a single dominant mechanism, but rather from the synergistic activation of multiple pathways that collectively optimise the process of endochondral ossification. Understanding the crosstalk among these pathways will be an important direction for future research. Similarly, the hDPSCs used in our in vitro model under osteogenic conditions generate a mixed cell population containing stem cell‐like cells, osteoprogenitors and mature osteoblasts. All of these cell types can express FABP5 and MMP9, making it difficult to attribute the observed effects solely to stem cell‐like differentiation. Future studies using lineage tracing or single‐cell transcriptomics are needed to dissect the respective contributions of stem cell‐like cells and osteoblasts to the observed phenotypes and to further clarify the cellular interaction mechanisms through which TPPU acts in the co‐culture system.

Although our study demonstrates that targeting sEH can regulate SCs involved in endochondral ossification, this study has several limitations. First, direct evidence linking EETs to SC regulation remains limited. The observed reduction in growth plate thickness may reflect accelerated cartilage‐to‐bone turnover rather than premature closure, yet long‐term follow‐up data are lacking. Previous studies have reported minimal toxicity of TPPU at comparable doses, supporting the safety of the current treatment regimen; however, comprehensive long‐term safety evaluations are still required for clinical translation. Furthermore, the lack of validation in pathological models such as arthritis and osteoporosis also limits the clinical translational significance of this study.

## 5 Conclusion

Our study demonstrates that TPPU enhances SCs' activity to promote long bone growth and fracture healing through HIF‐1α‐Notch signalling between endothelial and mesenchymal cells. The pharmacological inhibition of sEH may hold therapeutic potential for bone disorders by concurrently stimulating cartilage resorption and new bone formation, thereby supporting skeletal growth and repair.

## Author Contributions

F.W. and J.Z. designed and directed the study; W.C. and L.L. conducted and analysed the cellular and animal experiments; X.W. directed the animal experiments; J.L. and Q.S. carried out the animal and histological experiments; J.Z., X.S., X.C. and J.Z. were included in the data compilation. F.W. drafted and revised the manuscript. All authors read and approved the final manuscript.

## Funding

This work was supported by the National Natural Science Foundation of China (81771032) and Basic Scientific Research Project of Educational Department of Liaoning Province (LJKFZ20220249).

## Ethics Statement

The animal care and experimental protocols were approved by the Animal Care and Use Committee of Dalian Medical University (No. AEE23141). The isolation and culture of hDPSCs were approved by the Ethics Committee of Stomatological Hospital of Dalian Medical University (Approval No. 2022001). All participants gave their written informed consent.

## Conflicts of Interest

The authors declare no conflicts of interest.

## Supporting information


**Table S1:** Primer sequences for quantitative real‐time PCR.
**Table S2:** Target sequence of siRNA.
**Figure S1:** The effect of TPPU on long bone growth.
**Figure S2:** The FABP5^+^ cells at postnatal Day 0 or Day 7 in long bone.
**Figure S3:** The effects of TPPU on HUVECs or hDPSCs alone.
**Figure S4:** HIF‐1α knockdown in HUVECs.

**Figure S5:** Uncropped blot of Figure 2B for FABP5 and MMP9.
**Figure S6:** Uncropped blot of Figure 3B for DLL4 and NOTCH1.
**Figure S7:** Uncropped blot of Figure 4B for DLL4 NOTCH1 MMP9 and FABP5.
**Figure S8:** Uncropped blot of Figure 5A for HIF‐1α.
**Figure S9:** Uncropped blot of Figure 5C for HIF‐1α and DLL4.
**Figure S10:** Uncropped blot of Figure 5C for NOTCH1 and FABP5.
**Figure S11:** Uncropped blot of Figure S3A for DLL4 NOTCH1 and FABP5.
**Figure S12:** Uncropped blot of Figure S3D for NOTCH1 MMP9 and FABP5
**Figure S13:** Uncropped blot of Figure S4B for HIF‐1α.

## Data Availability

The data that support the findings of this study are available from the corresponding author upon reasonable request.
