# Supplementary material for: Soluble Epoxide Hydrolase Inhibition Regulates Septoclast Activity to Promote Long Bone Growth and Fracture Healing by Enhancing Endothelial‐to‐Mesenchymal Notch Signalling
Source: Cell Prolif. 2026 Jun 15;59(11):e70249. doi: 10.1111/cpr.70249 (PMC13325645; doi:10.1111/cpr.70249)
Supplement: Supplementary file 1 [file CPR-59-e70249-s001.docx]

**Uncropped full-length gels and blots.**

Figure S5

Uncropped blot of Fig. 2B for FABP5 and MMP9

Figure S6

Uncropped blot of Fig. 3B for DLL4 and NOTCH1

Figure S7

Uncropped blot of Fig. 4B for DLL4 NOTCH1 MMP9 and FABP5

Figure S8

Uncropped blot of Fig. 5A for HIF-1α

Figure S9

Uncropped blot of Fig. 5C for HIF-1α and DLL4

Figure S10

Uncropped blot of Fig. 5C for NOTCH1 and FABP5

Figure S11

Uncropped blot of Fig. S3A for DLL4 NOTCH1 and FABP5

Figure S12

Uncropped blot of Fig. S3D for NOTCH1 MMP9 and FABP5

Figure S13

Uncropped blot of Fig. S4B for HIF-1α
