# Supplementary material for: Soluble Epoxide Hydrolase Inhibition Regulates Septoclast Activity to Promote Long Bone Growth and Fracture Healing by Enhancing Endothelial‐to‐Mesenchymal Notch Signalling
Source: Cell Prolif. 2026 Jun 15:e70249. Online ahead of print. doi: 10.1111/cpr.70249 (PMC13325645; doi:10.1111/cpr.70249)
Supplement: Supplementary file 1 — Table S1: Primer sequences for quantitative real‐time PCR. Table S2: Target sequence of siRNA. Figure S1: The effect of TPPU on long bone growth. Figure S2: The FABP5+ cells at postnatal Day 0 or Day 7 in long bone. Figure S3: The effects of TPPU on HUVECs or hDPSCs alone. Figure S4: HIF‐1α knockdown in HUVECs. [file CPR-9999-e70249-s002.docx]

**Table S1 Primer sequences for quantitative real-time PCR**

| **Genes** | **Primer-Forward** | **Primer-Reverse** |
| --- | --- | --- |
| ***FABP5*** | AGCAGCTGGAAGGAAGATGC | CTGATGCTGAACCAATGCAC |
| ***GAPDH*** | GGAGCGAGATCCCTCCAAAAT | GGCTGTTGTCATACTTCTCATGG |
| ***DLL4*** | GCCCTTCAATTTCACCTGGC | CAATAACCAGTTCTGACCCACAG |
| ***MMP9*** | TCCCTGGAGACCTGAGAACC | GGCAAGTCTTCCGAGTAGTTT |
| ***NOTCH1*** | CCGCAGTTGTGCTCCTGAA | ACCTTGGCGGTCTCGTAGCT |

**Table S2 Target sequence of siRNA**

| **siRNA target genes** | **Target sequence** |
| --- | --- |
| ***sh-HIF-1α*** | 5’- CCAGCAGACUCAAAUACAATT -3’ |
| ***sh-NC*** | 5’- UUCUCCGAACGUGUCACGUTT -3’ |

**
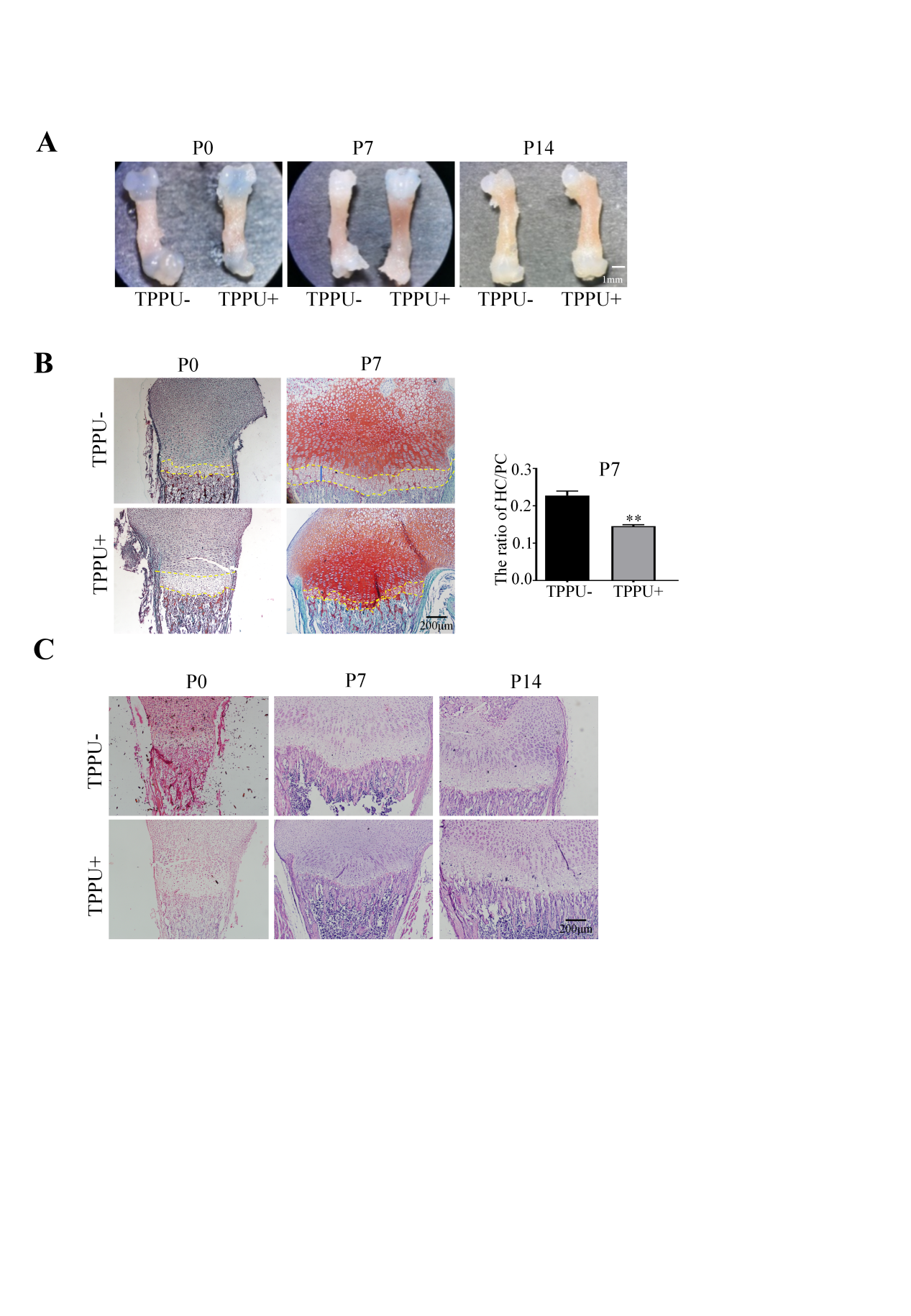
**

**Figure S1│The effect of TPPU on long bone growth**

(A) Representative images of femurs from pups in the TPPU- and TPPU+ groups from postnatal day 0, day 7, and day 14 pups. Scale bar=1 mm. (B) Safranin O-Fast Green staining of femurs from postnatal day 0 and day 7 pups. Quantitative analysis of the ratio of HC/PC. The HC and PC regions were measured from longitudinal sections based on morphological criteria. (C) H&E staining of femurs from postnatal day 0, day 7, and day 14 pups. Scale bar=200 μm. **P* < 0.05. n=6.


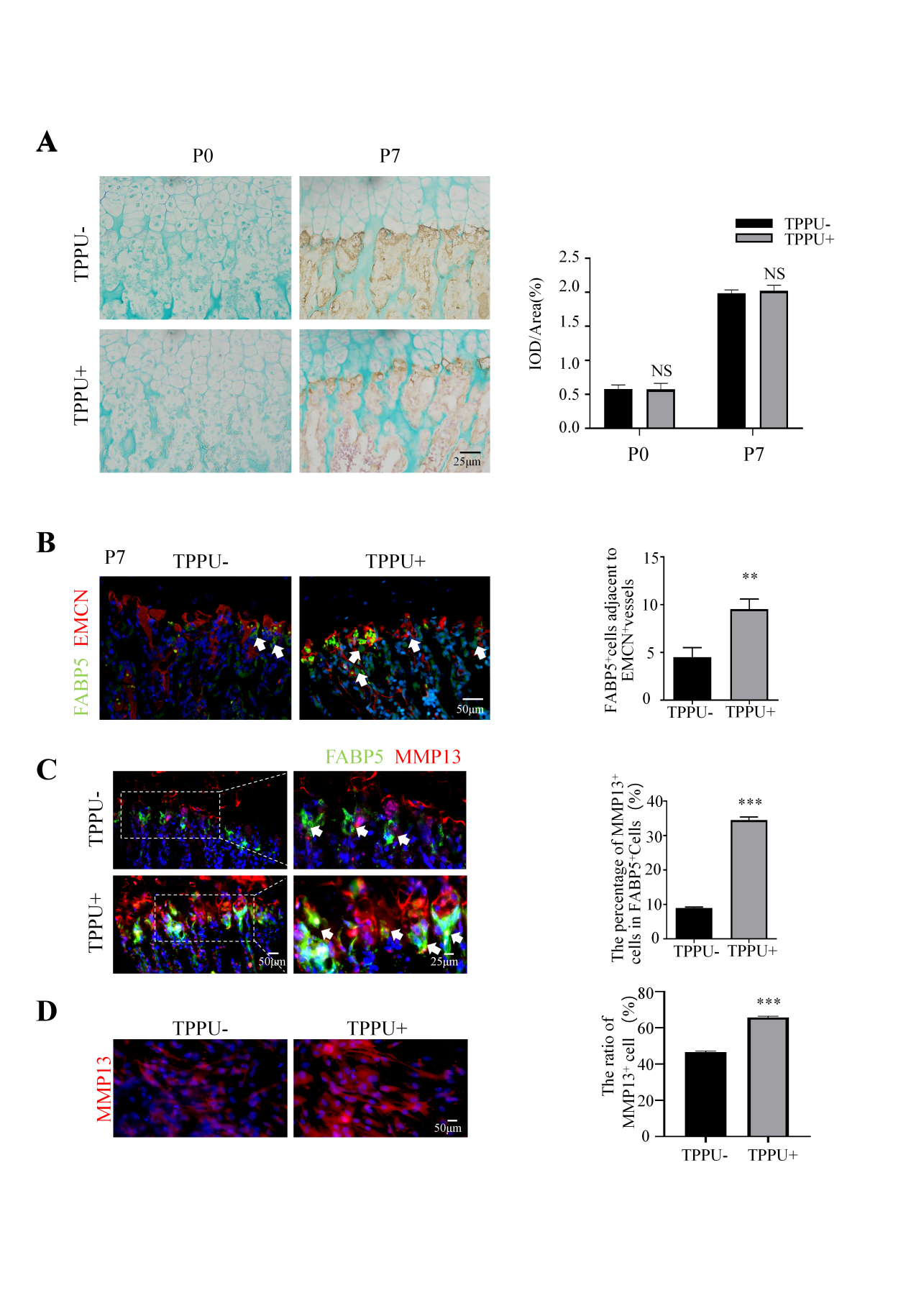


**Figure S2│The FABP5^+^ cells at postnatal day 0 or day 7 in long bone**

(A) IHC and Alcian blue staining revealed expression of FABP5 in the metaphysis adjacent to the growth plate in postnatal day 0 and day 7 pups. Scale bar=25 μm. (B) FABP5^+^(green) septoclasts (white arrows) near EMCN^+^ vessel buds (red) in the metaphysis of postnatal day 7 pups. (C) Immunofluorescence staining of FABP5 (green) and MMP13 (red) in the metaphyseal region of 2-week-old neonatal mice. The percentage of MMP13^+^ cells among FABP5^+^ cells is shown on the right. (D) Immunofluorescence staining of MMP13 (red) in the co-culture system of HUVECs and hDPSCs. Quantification of the proportion of MMP13^+^ cells is shown on the right.

The scale bar=50 μm. **P* < 0.05, ***P* < 0.01. n=6.


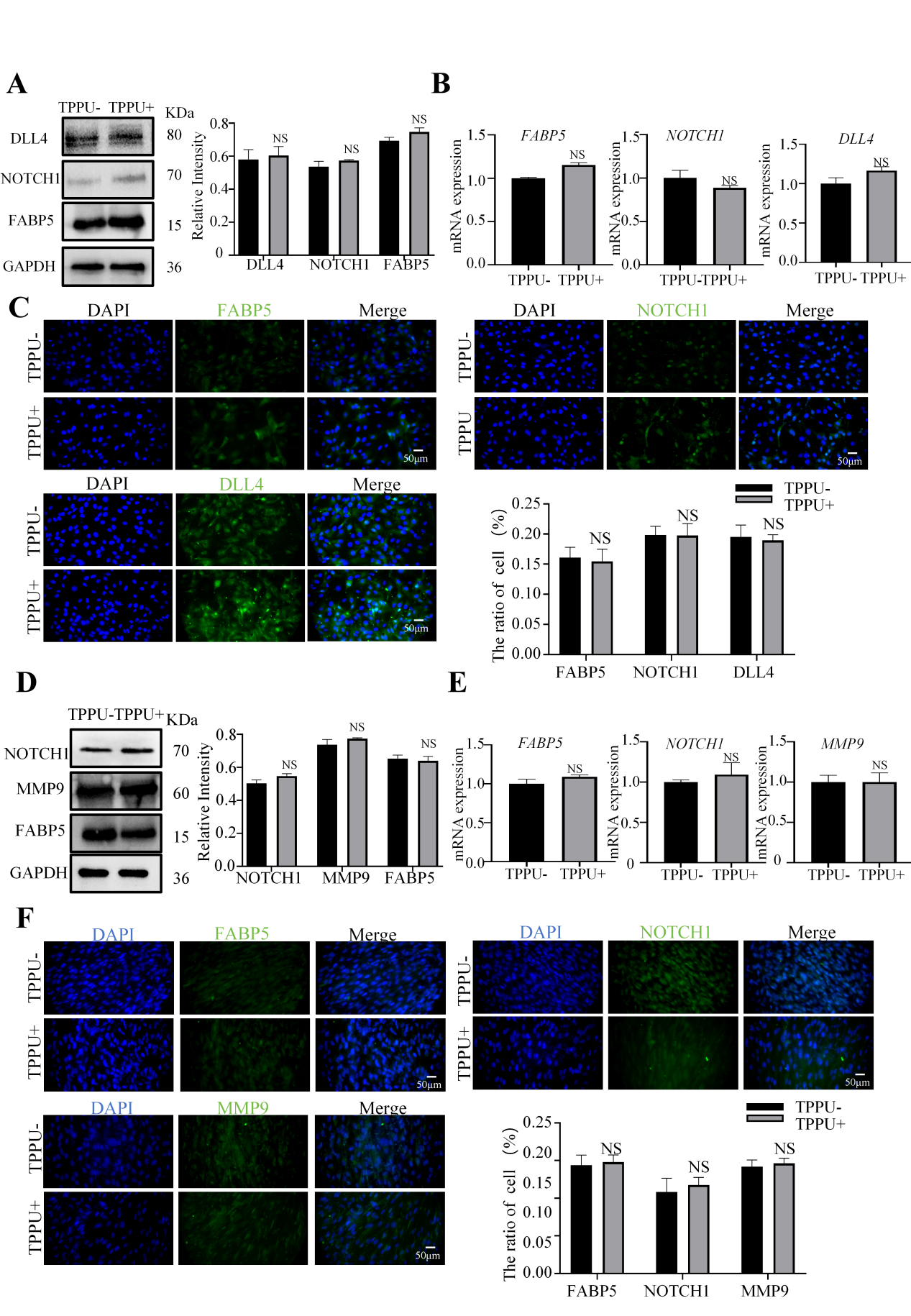


**Figure S3│The effects of TPPU on HUVECs or hDPSCs alone**

(A, B) RT-qPCR and Western Blot analysis showing FABP5 NOTCH1, and DLL4 expression in cultured HUVECs alone (3 samples of three independent experiments). Full-length blots/gels were presented in Supplementary Fig. 11. (C) Representative image showing FABP5, NOTCH1 and DLL4 (green) in HUVECs alone (3 samples of three independent experiments). Scale bar=50 μm. (D, E) RT-qPCR and Western Blot analysis showing FABP5, NOTCH1 and MMP9 expression in cultured hDPSCs alone (3 samples of three independent experiments). Full-length blots/gels were presented in Supplementary Fig. 12. (F) Representative image showing FABP5, NOTCH1 and MMP9 (green) in DPSCs alone (3 samples of three independent experiments). Scale bar=50 μm. n= 3.


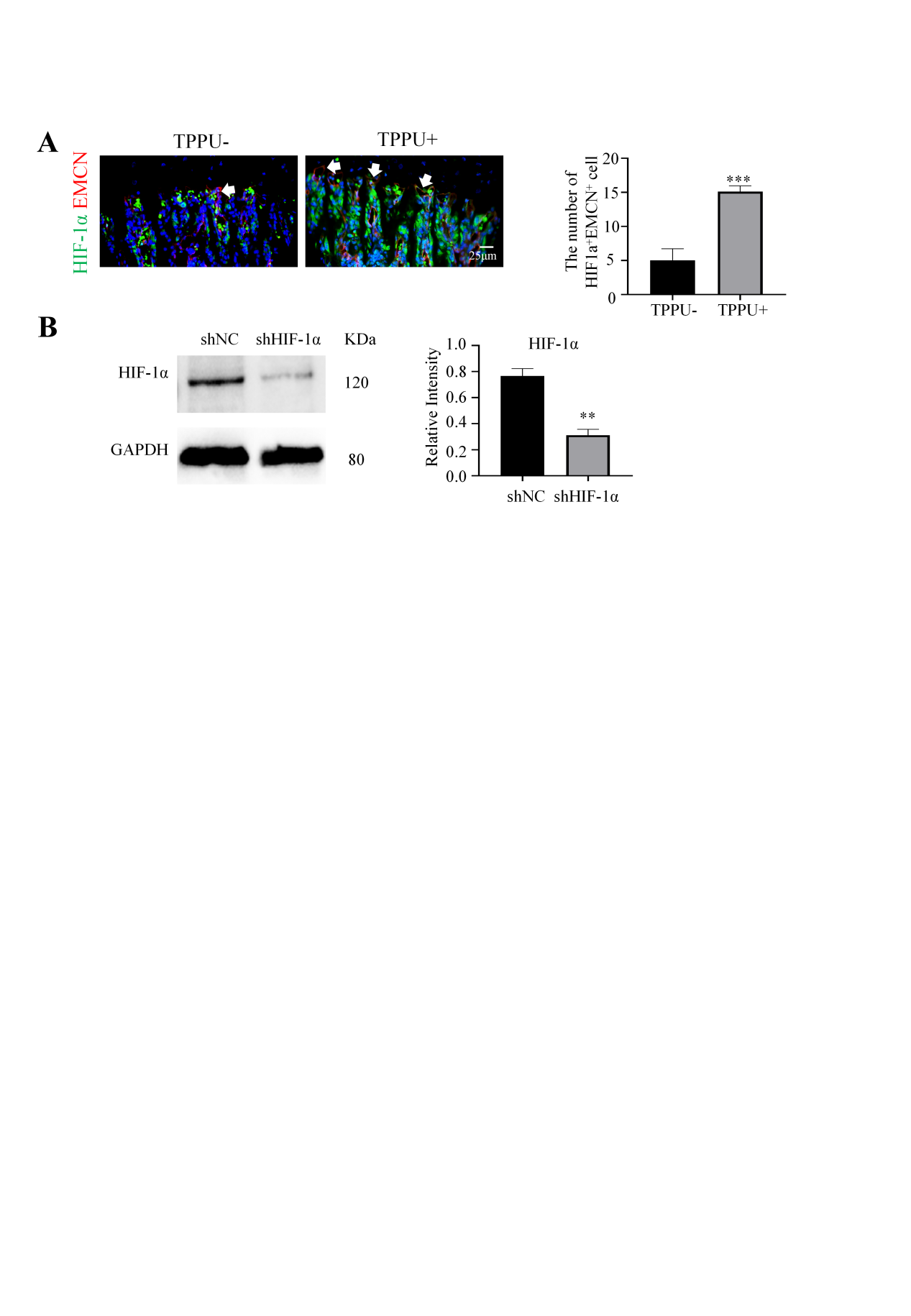


**Figure S4│HIF-1α knockdown in HUVECs**

(A) HIF-1α (green) co-located with EMCN^+^ vessel buds (red) (white arrows). The left scale bar=50 μm, the right scale bar=25 μm. **P* < 0.05, ***P* < 0.01. n= 5. (B)Western blot analysis showing HIF-1α knockdown efficiency in HUVECs. Full-length blots/gels were presented in Supplementary Fig. 13. ***P* < 0.01, ****P* < 0.001.
