# Supplementary material for: Soluble Epoxide Hydrolase Inhibition Regulates Septoclast Activity to Promote Long Bone Growth and Fracture Healing by Enhancing Endothelial‐to‐Mesenchymal Notch Signalling
Source: Cell Prolif. 2026 Jun 15:e70249. Online ahead of print. doi: 10.1111/cpr.70249 (PMC13325645; doi:10.1111/cpr.70249)
Supplement: Supplementary file 2 — Figure S5: Uncropped blot of Figure 2B for FABP5 and MMP9. Figure S6: Uncropped blot of Figure 3B for DLL4 and NOTCH1. Figure S7: Uncropped blot of Figure 4B for DLL4 NOTCH1 MMP9 and FABP5. Figure S8: Uncropped blot of Figure 5A for HIF‐1α. Figure S9: Uncropped blot of Figure 5C for HIF‐1α and DLL4. Figure S10: Uncropped blot of Figure 5C for NOTCH1 and FABP5. Figure S11: Uncropped blot of Figure S3A for DLL4 NOTCH1 and FABP5. Figure S12: Uncropped blot of Figure S3D for NOTCH1 MMP9 and FABP5 Figure S13: Uncropped blot of Figure S4B for HIF‐1α. [file CPR-9999-e70249-s001.docx]

**Uncropped full-length gels and blots.**

Figure S5

Uncropped blot of Fig. 2B for FABP5 and MMP9


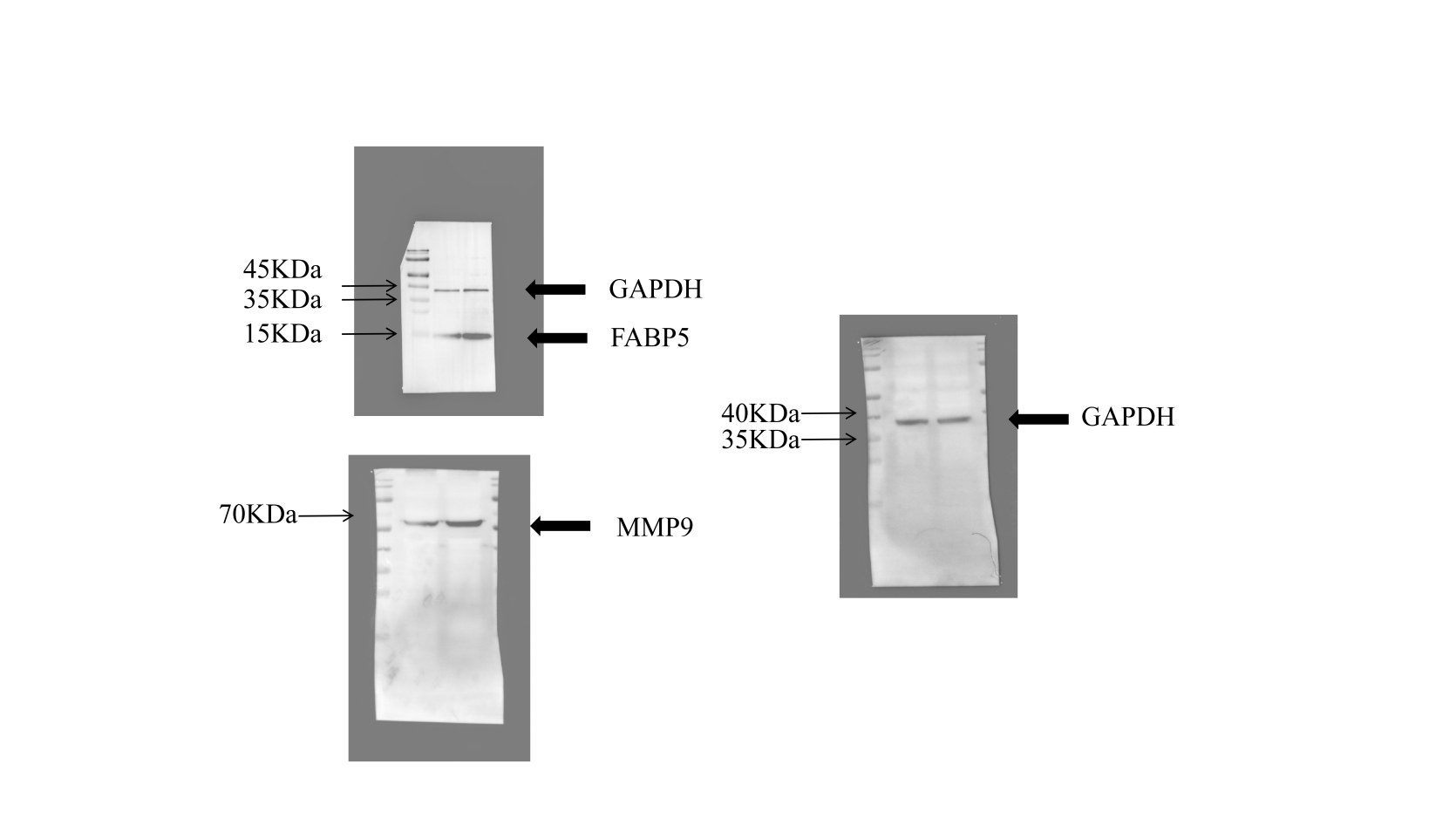


Figure S6

Uncropped blot of Fig. 3B for DLL4 and NOTCH1


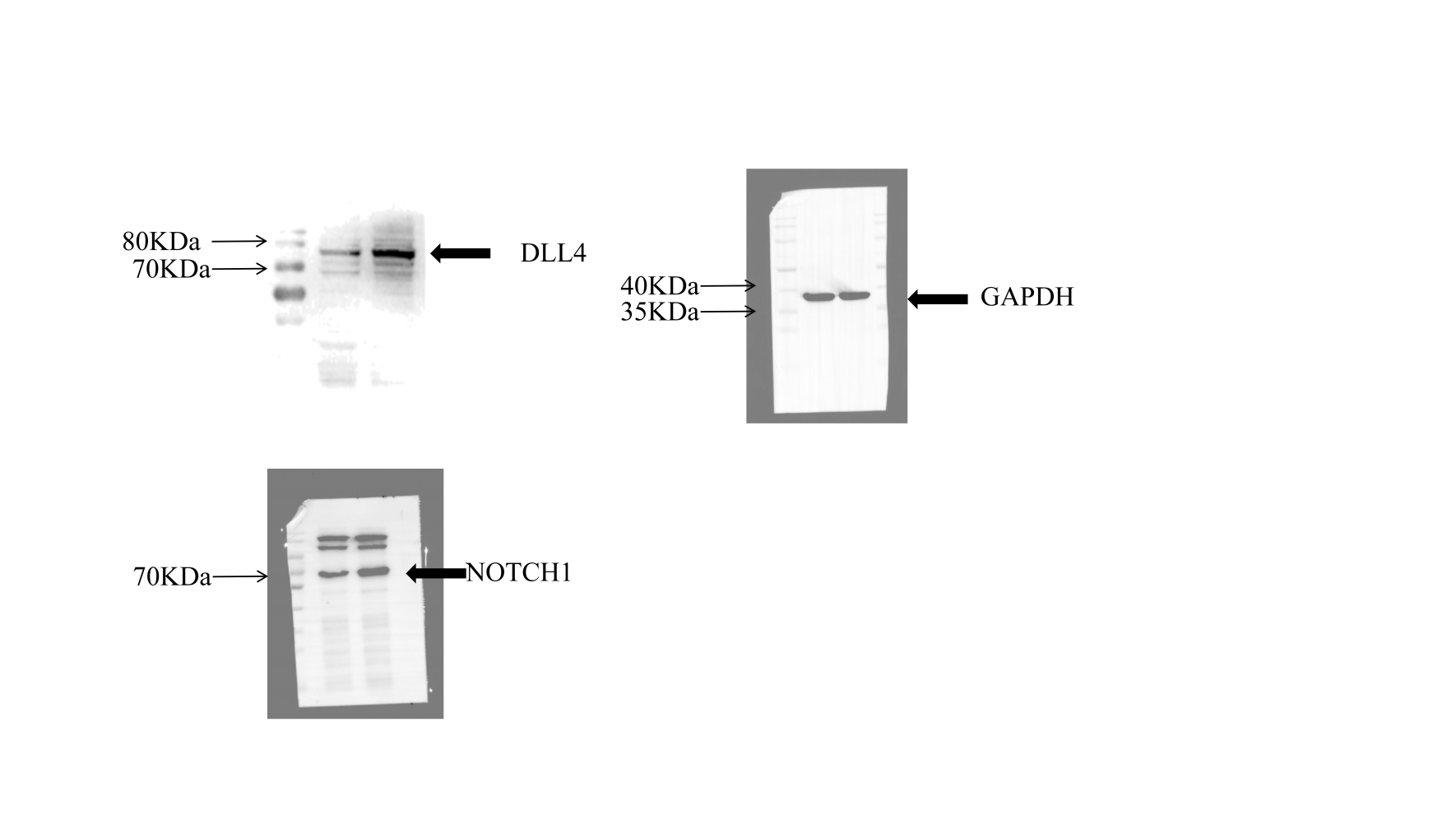


Figure S7

Uncropped blot of Fig. 4B for DLL4 NOTCH1 MMP9 and FABP5


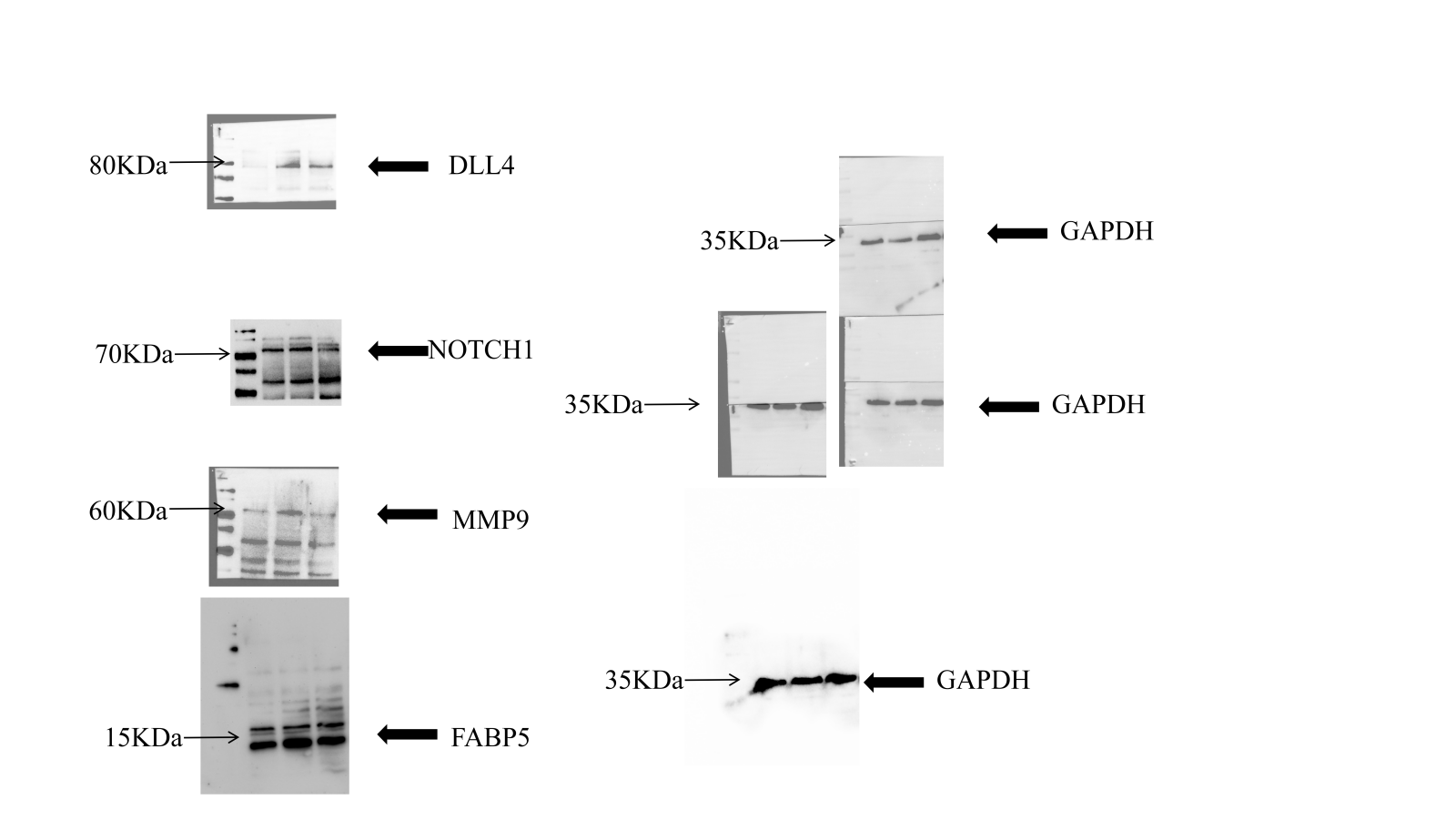


Figure S8

Uncropped blot of Fig. 5A for HIF-1α


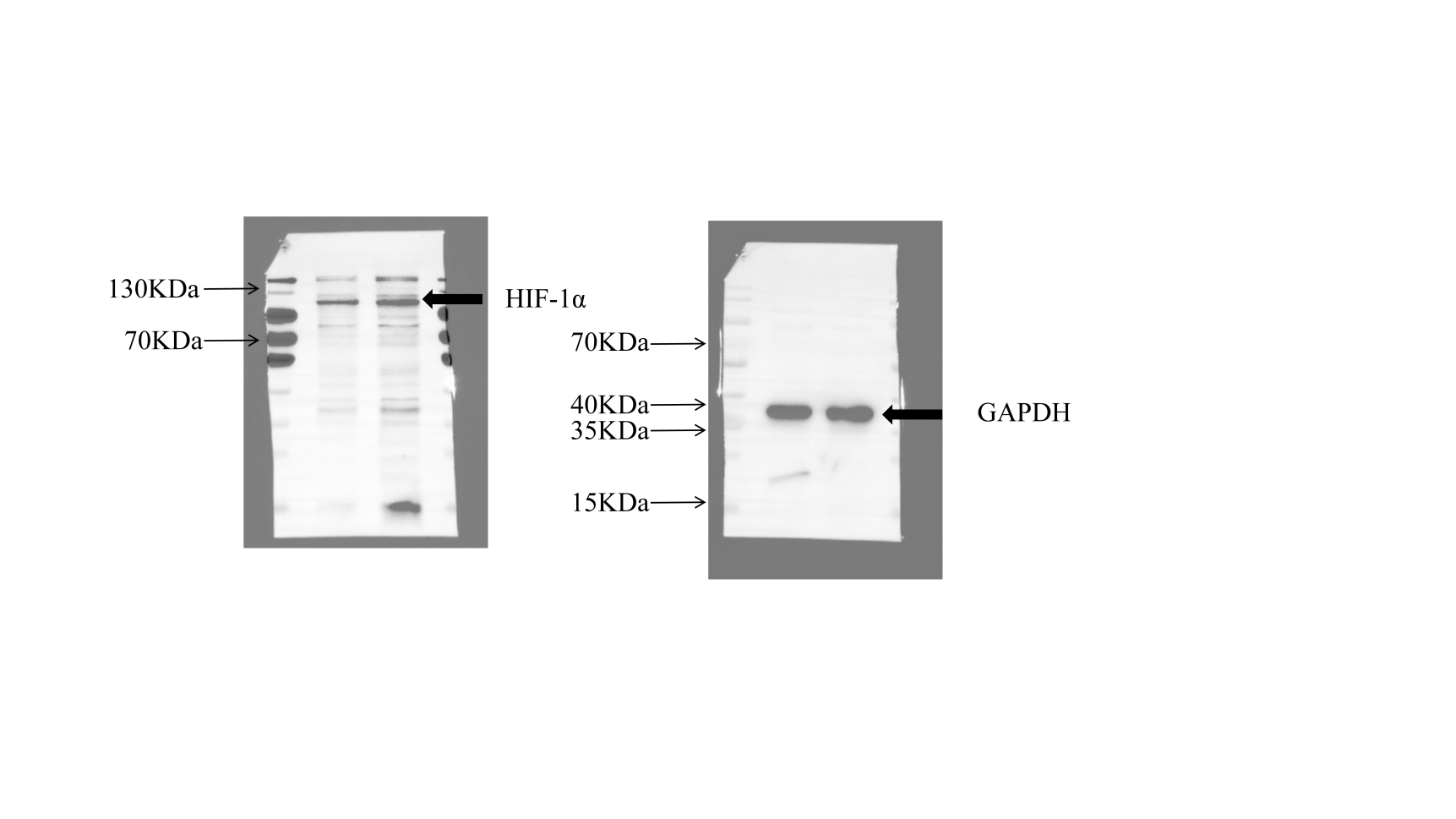


Figure S9

Uncropped blot of Fig. 5C for HIF-1α and DLL4


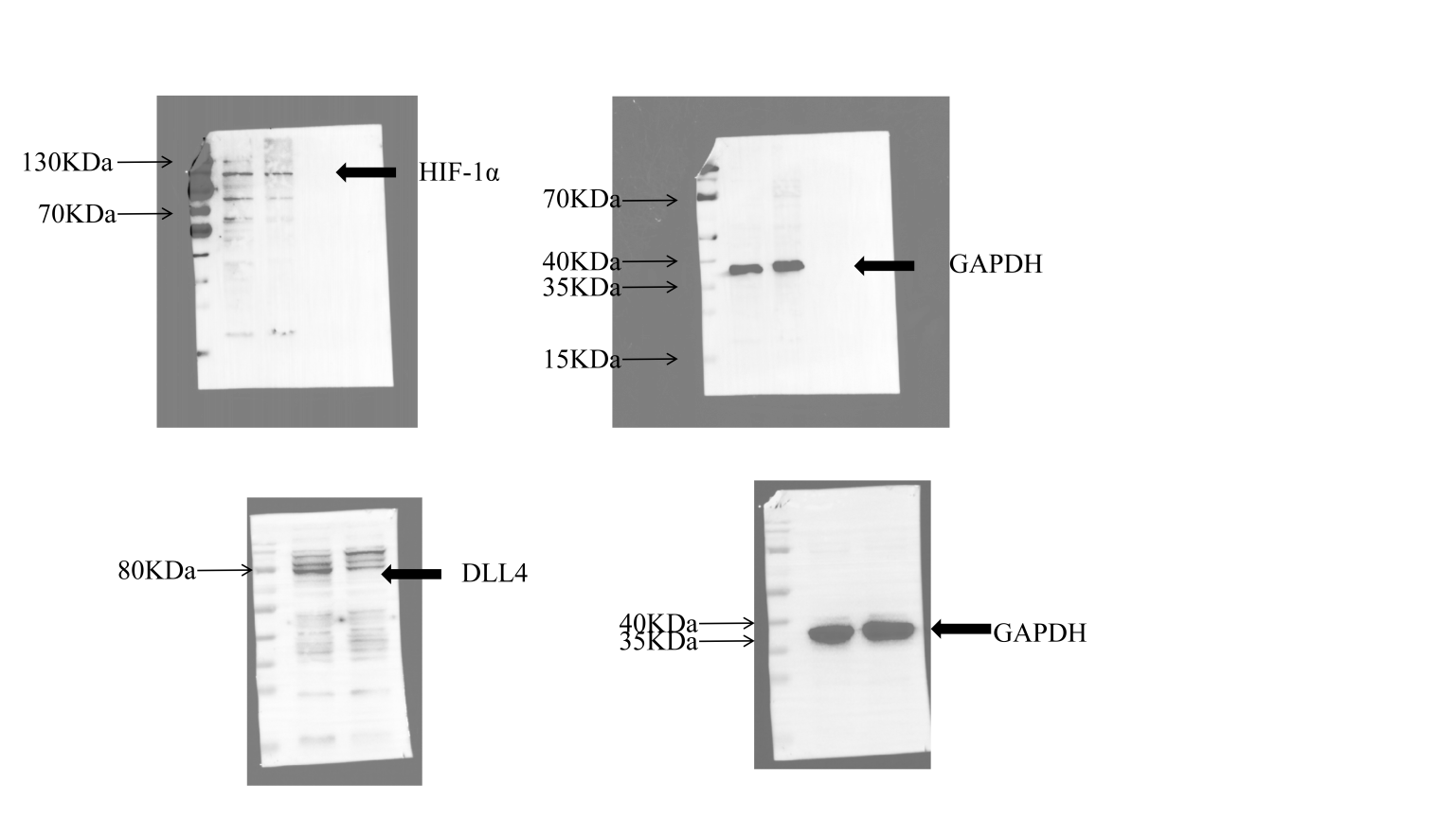


Figure S10

Uncropped blot of Fig. 5C for NOTCH1 and FABP5


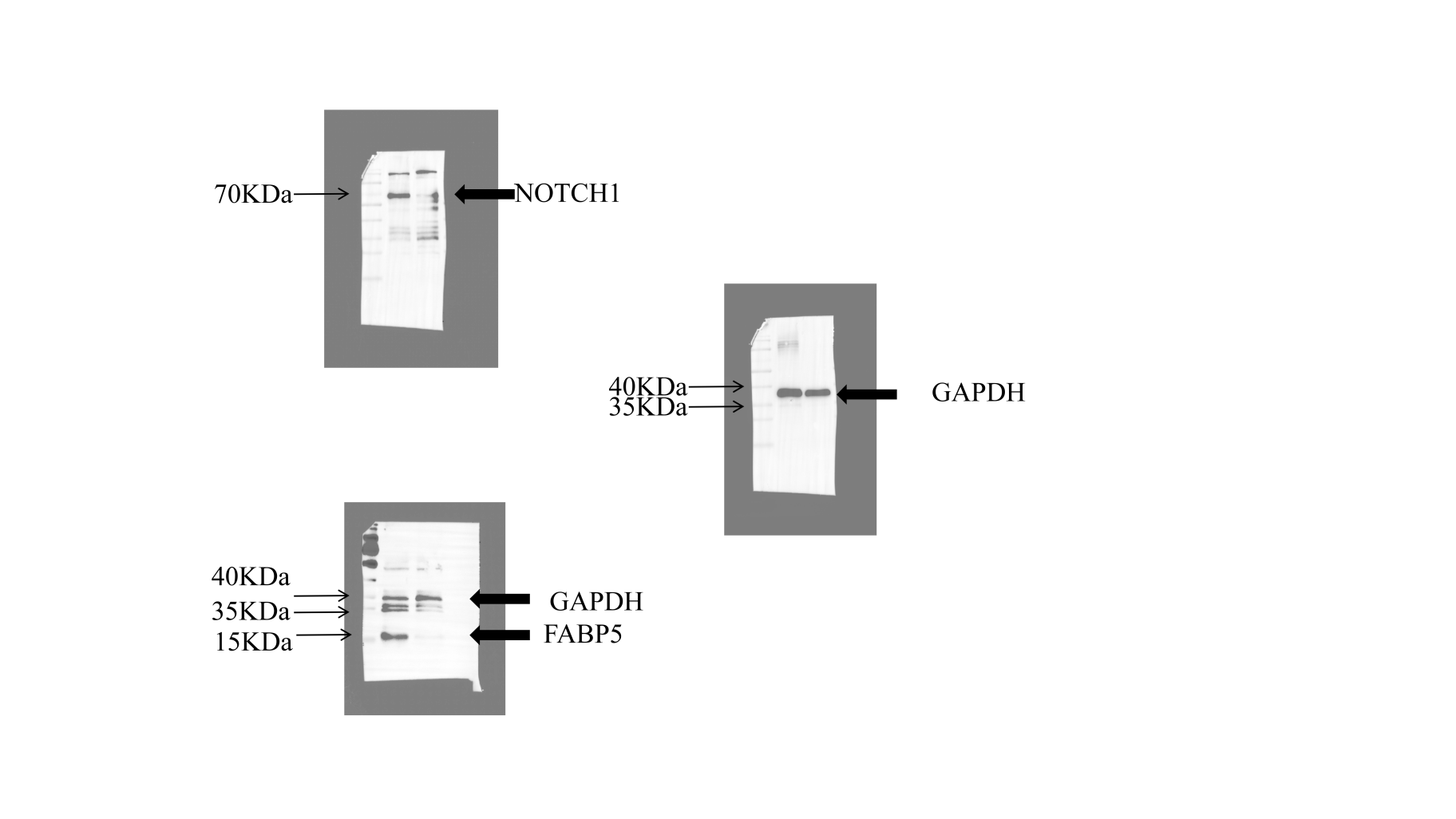


Figure S11

Uncropped blot of Fig. S3A for DLL4 NOTCH1 and FABP5


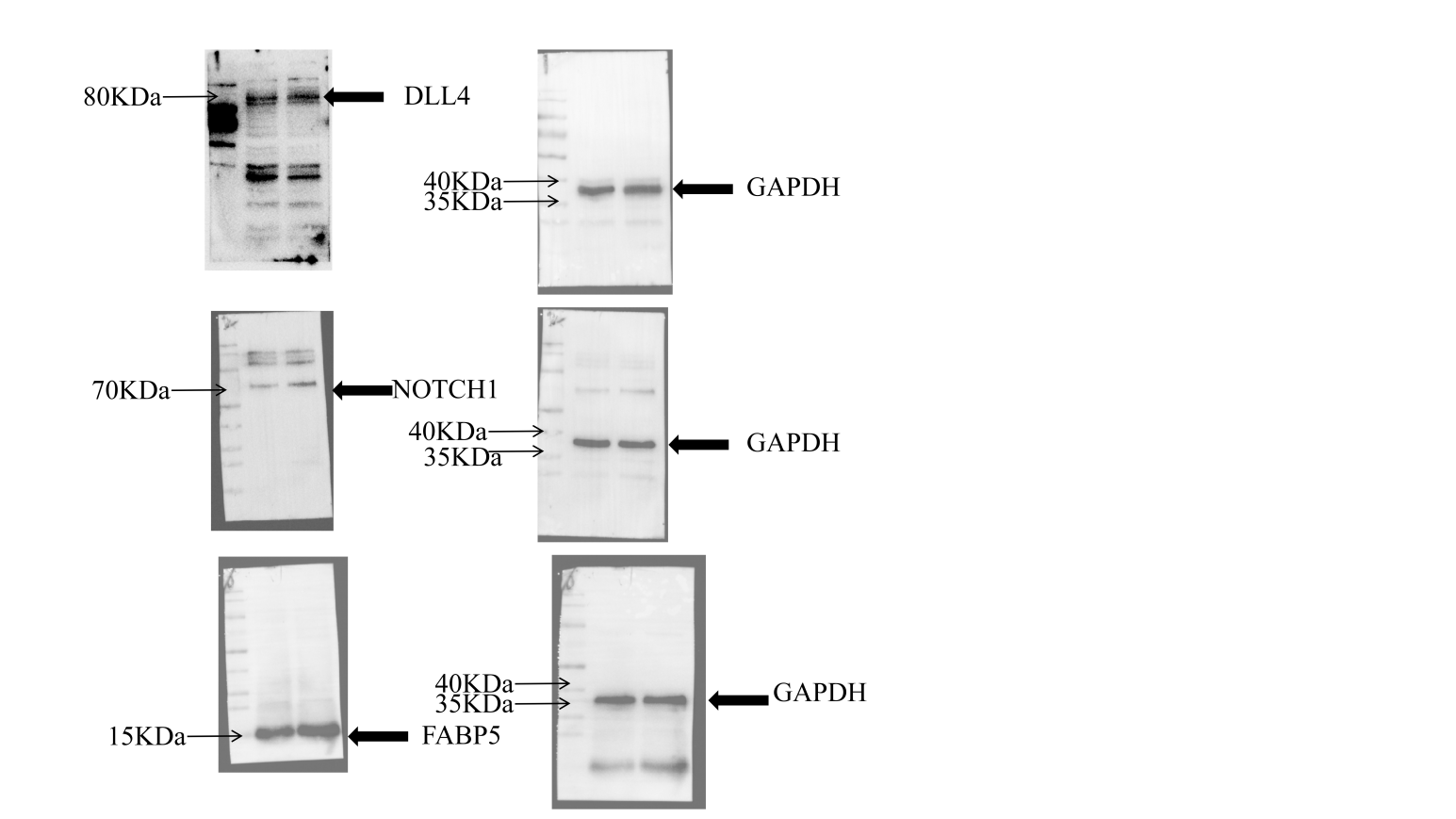


Figure S12

Uncropped blot of Fig. S3D for NOTCH1 MMP9 and FABP5


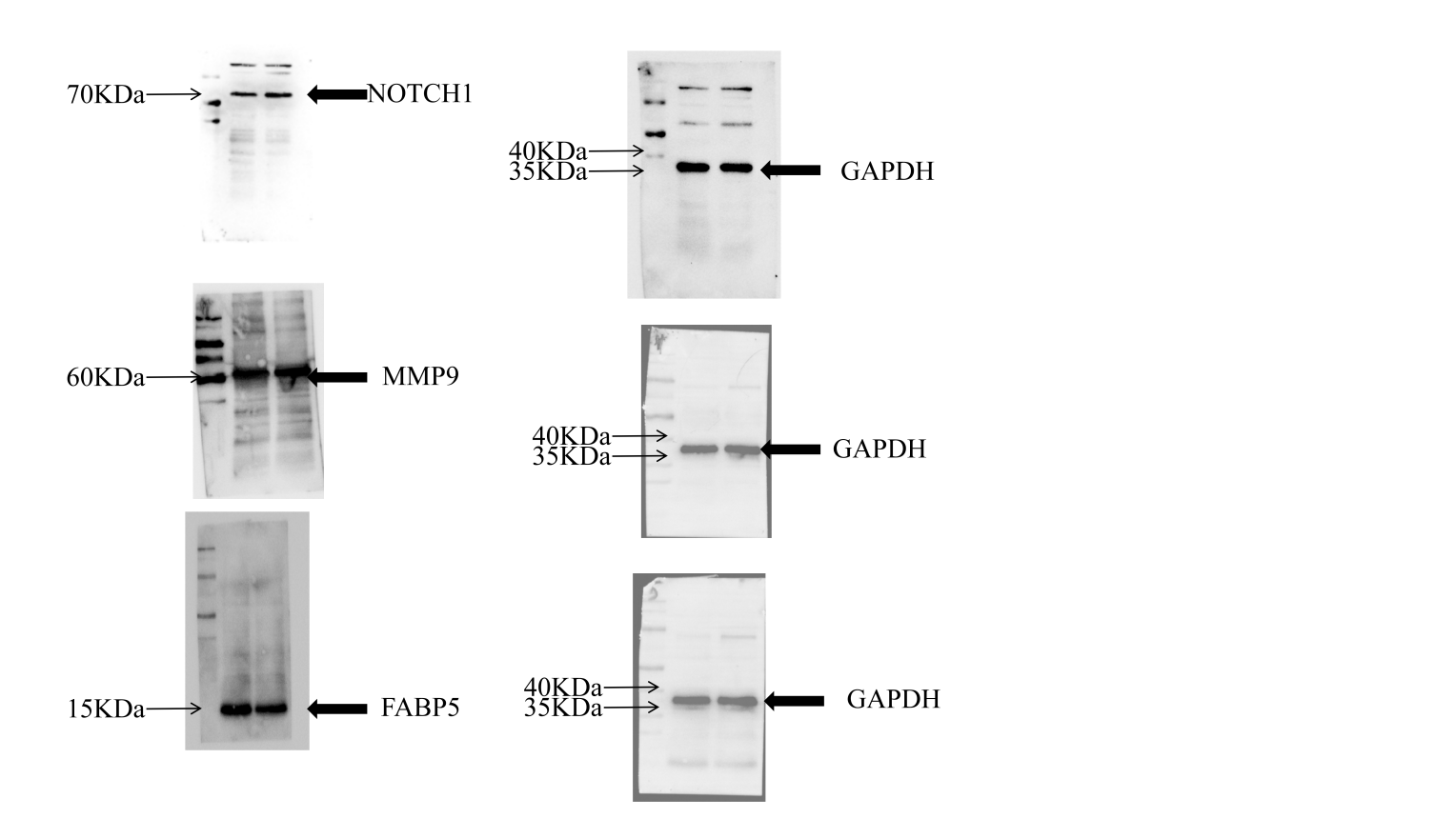


Figure S13

Uncropped blot of Fig. S4B for HIF-1α

**
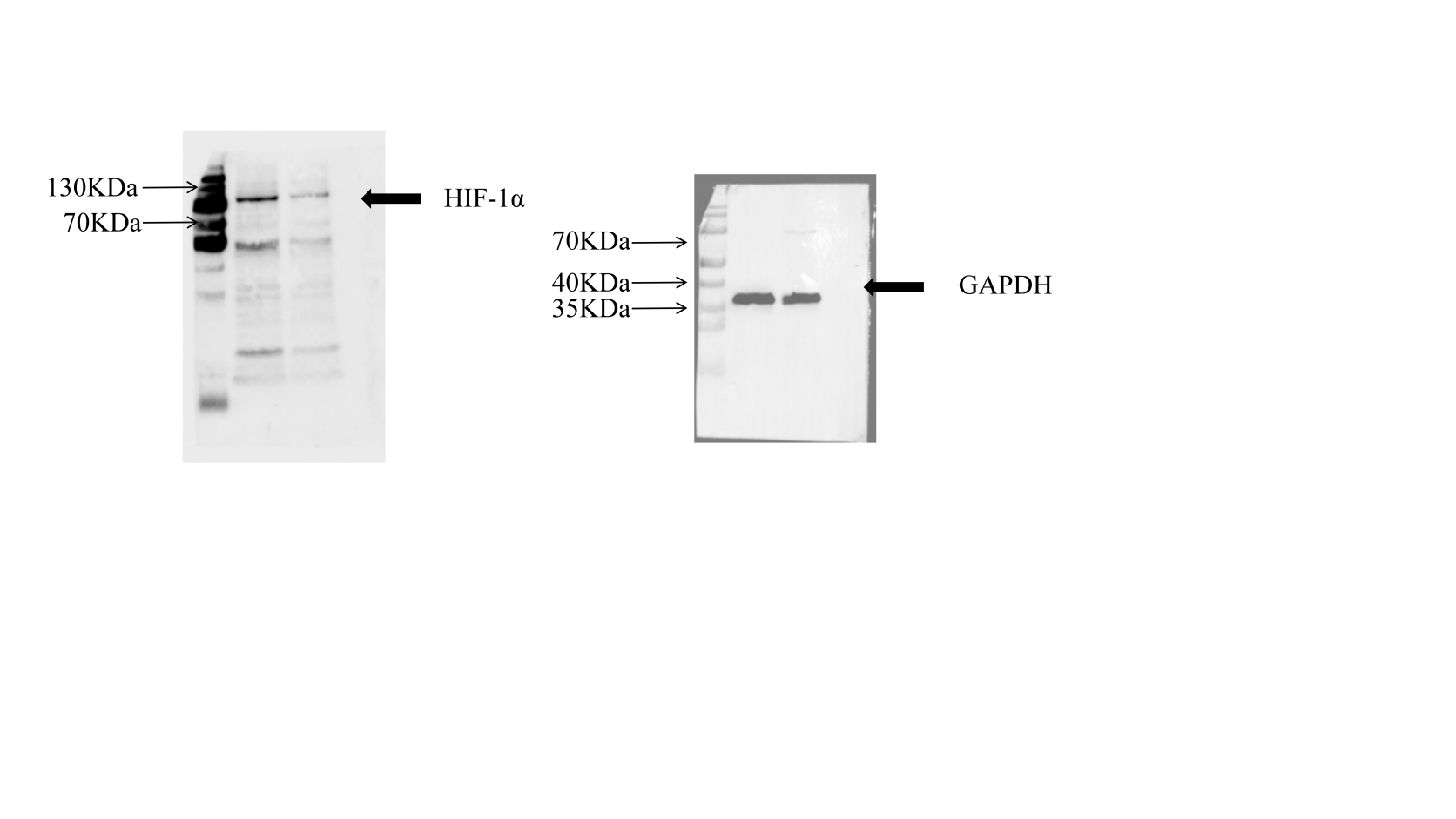
**
